# Strands in a Web: Free Energies of Select Prebiotic Reactions with Sugars, Nitriles and Azoles

**DOI:** 10.3390/life16091531

**Published:** 2026-09-15

**Authors:** Jeremy Kua, Alexandra N. Mateescu

**Affiliations:** Department of Chemistry & Biochemistry, University of San Diego, San Diego, CA 92110, USA

**Keywords:** origins of life, prebiotic chemistry, sugars, HCN, cyanamide, azoles, cysteine

## Abstract

Using computational chemistry, we determined the free energy changes for the reactions of C_2_ and C_3_ sugars with HCN and NH_2_CN to form amino acids and azoles respectively. Amino acids and azoles may further react with the initial sugars to produce more complex molecules, adding to the complex web of prebiotic chemistry with its myriad species. For the reactions we have selected, our calculations provide a guide, albeit incomplete, of which pathways are thermodynamically and/or kinetically favorable and which species might be observed starting from just a handful of simple reactants. The reactions examined are strands in a web, connecting different sectors of prebiotic chemistry to the larger network of origins-of-life chemistry.

## 1. Introduction

The riddle of life’s chemical origins is no longer about synthesizing the organic compounds found in extant biochemistry from simpler smaller molecules. Rather, the confounding question is why, out of the large range of compounds produced in prebiotic chemical syntheses, living systems seem to subsist on a much narrower subset. Evaluating the thermodynamics and kinetics of such reactions may provide a clue.

The sugar-producing formose system is a good example of how C–C bonds are formed via simple aldol reactions. Starting with the C_1_ species formaldehyde (CH_2_O), self-oligomerization generates a large diversity of sugars (CH_2_O)_n_ [1]. Glucose and ribose are the most familiar of the C_6_ and C_5_ sugars respectively, and while life uses these sugars (and several others), most of the formose products are not found naturally in living cells. In addition to sugars, the formose system also produces carboxylic acids and polyols via Cannizzaro reactions; some are found in extant biochemistry, but not most.

The basic formose reaction mechanism has been worked out experimentally [2,3,4]. Its core autocatalytic cycle consists of just three steps involving (CH_2_O)_n_ molecules (*n* = 1–4) and we previously constructed a free energy map of this system [5]. The linchpin molecule is the C_2_ sugar glycolaldehyde (C_2_H_4_O_2_). The first two steps involve kinetically accessible C–C bond formations: an aldol addition of CH_2_O to C_2_ leads to the C_3_ sugar glyceraldehyde, and a second aldol addition of CH_2_O converts the C_3_ to the C_4_ tetrose. The third reaction is a retroaldol conversion of C_4_ back to two equivalents of C_2_. Thus, the net reaction of this cycle is C_1_ + C_1_ → C_2_, but it avoids the kinetically inaccessible direct C–C bond formation between two C_1_ species. For the formose system to be facile in building up larger sugars, at least a small amount of a C_2_ (or larger) sugar has to be present in the reaction mixture for facile aldol addition.

One way to avoid the challenge of forming a C–C bond between two C_1_ compounds with electrophilic carbons is for one of them to have a nucleophilic carbon. HCN is a plausible prebiotic candidate [6,7] and readily reacts with CH_2_O (in aqueous solution and with NH_3_ present) to form the amino acid glycine via the Strecker mechanism. In his famous 1953 Miller experiment [8], CH_2_O and HCN were produced in situ from the starting gases, leading to the formation of glycine and glycolic acid, albeit in low yield because a plethora of other compounds are produced in the final complex mixture [9], typical of such prebiotic syntheses. We previously calculated the free energies of the C_2_ compounds formed after C–C bond formation between CH_2_O and HCN, thus providing a thermodynamic and kinetic map of which species are expected to be observed and which pathways are feasible [10]. This study consisted of just 39 compounds and 50 reactions, yet represents only a small portion of a much larger reaction network.

**Figure 1:**
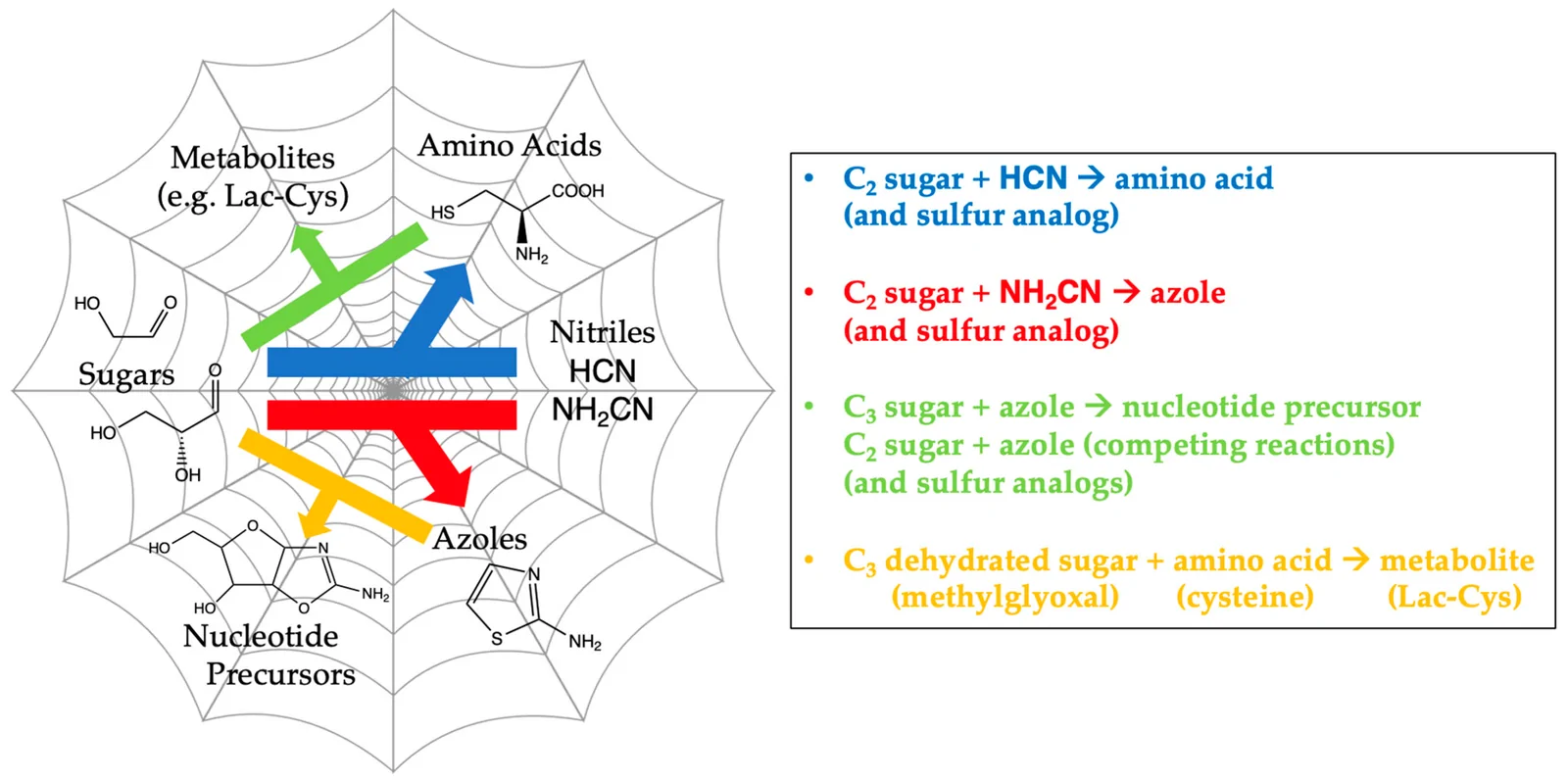
Strands in a web connecting the compounds and reactions examined in this work.

An overall view of our present work is shown in Figure 1. The web shown depicts a subset of compounds relevant to prebiotic chemistry; the sectors are classes of compounds (amino acids, nitriles, sugars, etc.). We explore four strands in this web, each connecting two sectors to a third. The first strand, represented by the blue arrow in Figure 1, combines sugars and nitriles to form amino acids. In particular, we calculate the optimal free energy pathway for reacting the C_2_ sugar glycolaldehyde with HCN to form the amino acid serine. Because our group has studied the thiol analogs of the formose reaction [11], we also examine the parallel reaction of mercaptoaldehyde (the thiol analog of the formose cycle linchpin) to form cysteine.

Nitriles have become ubiquitous in prebiotic chemistry because of their utility in producing a variety of the biomolecules in extant life, from early work synthesizing purine nucleobases [12] to recent studies generating metabolite analogs of the tricarboxylic acid cycle [13], and even coupling nucleotide precursor synthesis to the formose reaction [14]. Cyanamide (NH_2_CN) has been proposed as a prebiotic precursor [15] and utilized extensively in prebiotic nucleotide syntheses [7]. Thus, the second strand, represented by the red arrow in Figure 1, examines the reaction of the same C_2_ sugars (glycolaldehyde and mercaptoaldehyde) with NH_2_CN to form their corresponding azoles.

The third strand (yellow arrow in Figure 1) explores the reaction of the azoles with both the C_2_ and C_3_ (glyceraldehyde) sugars. Experimentally this has proved a successful route to aminooxazolines, precursors to the prebiotic synthesis of nucleotides, as demonstrated by Sutherland, Powner and their colleagues [16,17,18,19]. Echoing the aldol additions in the formose reaction to yield new C–C bonds, the addition reaction of sugar aldehyde and azole is facilitated by the electrophilic enol-like moiety in the azole.

Our fourth and final strand (green arrow in Figure 1) examines the reaction of the amino acid cysteine with methylglyoxal (the dehydrate of the C_3_ sugar glyceraldehyde) to form the metabolite LacCys (an amide dimer of lactic acid and cysteine). We chose to focus on this reaction because there is very recent experimental work on this reaction in aqueous solution studying the role of the glyoxalase enzyme in catalyzing this reaction in extant life [20]. The experimental study postulated the existence of a thioester intermediate, which relates to our recent work examining the reaction of sugars reacting with thiols and undergoing internal disproportionation to form thioesters [21]. This meshes with De Duve’s proposed Thioester World [22] and experimental prebiotic reactions by Weber utilizing glyceraldehyde and a cysteine analog [23,24].

We present the calculated free energy pathways for these four strands in Section 3 along with their relevance to prebiotic chemistry. We utilize what we have learned from our calculations to elaborate previous experimental results, but also discuss what we cannot answer with our computational methodology. In particular, our protocol only works well for neutral species and does not account for the wider range of pH in potential prebiotic chemistry environments.

## 2. Computational Methods

Our computational protocol is similar to our recent work on the formose reaction [5] and its thiol analogs [11]. With our strands-in-a-web approach, this allows us to enlarge our free energy map by connecting more puzzle pieces using the same free energy scale. In this section, we recap our methodology so that the reader does not have to peruse a different article to determine this. Much of the text in this section (and the first two paragraphs of the next section) is reproduced from those two articles (also published in this journal) since we believe that the description is succinct yet sufficiently informative. Our calculated free energies using this quantum chemistry protocol showed good agreement with available experimental results both for CHO systems relevant to this study [25,26] and reactions involving HCN [27].

All quantum calculations were carried out with Q-Chem 6.2 [28]. Here are the details: The geometry of each molecule is optimized and its electronic energy calculated with the B3LYP density functional [29,30,31,32] including Grimme’s D3 dispersion correction [33] with the 6-311G** basis set. To maximize the probability of finding global minima, multiple conformers are generated and optimized. Each optimized structure is embedded in a continuum dielectric to calculate the aqueous solvation contribution to the free energy. While this does not provide a specific concentration, it assumes a dilute solution such that the electrostatic field generated by a neighboring solute molecule is effectively screened by a dielectric representing water using the SMD implicit solvent model [34].

Zero-point energy corrections are included, and we apply the standard temperature-dependent enthalpy correction term (for 298.15 K) from statistical mechanics by assuming translational and rotational corrections are a constant times *RT*, and that low frequency vibrational modes generally cancel out when calculating enthalpy differences. However, entropic corrections in aqueous solution are problematic [35,36,37]. Changes in free energy terms for translation and rotation are poorly defined in solution due to restricted complex motion, particularly as the size of the molecule increases (thus increasing its conformational entropy). Free energy corrections come from two different sources: thermal corrections and implicit solvent. Neither of these parameters is easily separable, nor do they constitute all the required parts of the free energy. We follow the approach of Deubel & Lau [38], assigning the solvation entropy of each species as *half* its gas-phase entropy (calculated using standard statistical mechanics approximations similar to the enthalpy calculations described above), based on proposals by Wertz [39] and Abraham [40] that upon dissolving in water, molecules lose a constant fraction (~0.5) of their entropy.

To estimate activation energies, transition states were optimized (closed-shell) by including several explicit water and/or catalytic molecules to facilitate proton transfer. All optimized transition states have one significant negative eigenvalue corresponding to the reaction coordinate, and each was checked to ensure it had the correct eigenvectors for bond breaking/forming connecting the transition state to its reactants and products. Several conformers are built and fully optimized, and for each reaction we only report the lowest calculated barriers with the optimum number of solvent molecules added to assist in proton transfer. In the majority of cases (but not all), including two additional explicit water molecules was optimal.

Our protocol performs well comparing our calculated equilibrium concentrations with experimental NMR results of the product mixture resulting from self-oligomerizing aqueous solutions of glycolaldehyde [25] and methylglyoxal [26]. Both these molecules are starting reactants for pathways in the present work. In addition, a comparison of our calculated free energies is in good agreement with thermodynamic databases of CHO-containing biomolecules [41]. Our relative Gibbs free energies in aqueous solution are typically within 0.5 kcal/mol compared to experiment. That being said, our protocol has systematic errors of 2–3 kcal/mol when calculating barriers involving carbonyl chemistry when compared to experimental results. Going to a higher level of theory does not improve the results [26] (also see Supplementary Materials), nor does using charged species; our protocol shows better agreement with experimental results for the neutral forms, e.g., using COOH rather than carboxylate for carboxylic acids [41]. Quantum chemistry is about error cancelation, and our protocol (with its foibles, including the simplistic entropy correction) has worked well even with a systematic error for activation barriers.

## 3. Results and Discussion

Throughout this paper, the thermodynamics of a chemical reaction will be denoted by ∆*G*. For transition states, kinetic barriers will be denoted by ∆*G*^‡^ and will refer to the forward barrier unless otherwise specified. Given our strands-in-a-web approach, a consistent set of reference compounds allows us to compare energies globally. In the figures without energy curves, *G*_rel_ values are found under each compound for local minima and in parenthesis next to an arrow for transition states. For a chemical reaction, ∆*G* = *G*_rel_(products) − *G*_rel_(reactants), and ∆*G*^‡^ compares *G*_rel_ of the transition state to either the reactants or products depending on whether the forward or reverse reaction is being discussed. The *G*_rel_ values for all compounds parsed into their separate electronic, enthalpic, entropic, and solvation components are provided in Supplementary Materials.

The *G*_rel_ values are calculated *relative* to five reference substances: CO_2_, H_2_O, H_2_, H_2_S, and NH_3_. The first four are the same reference compounds used in our prior thermodynamic map examining the formose cycle and its thiol analogs [11]. For nitrogen, we chose the simpler NH_3_ instead of HCN (which complicates the analysis given that it contains three elements). These five substances are assigned a *relative* free energy, *G*_rel_ of 0.0 kcal/mol. The *G*_rel_ values of all other species can be determined by calculating the change in free energy for *forming* the species, analogous to a free energy of formation. For example, the formation reaction of cysteine is written as

3 CO_2_ + 5 H_2_ + NH_3_ + H_2_S → C_3_H_7_NO_2_S + 4 H_2_O

Since the change in free energy of this reaction is −35.3 kcal/mol, we assign *G*_rel_(C_3_H_7_NO_2_S) = −35.3 kcal/mol. All non-reference state compounds have *G*_rel_ values calculated analogously. Note that the nitrile starting reactants HCN and NH_2_CN have *G*_rel_ values of +9.2 and +17.1 kcal/mol respectively according to this protocol.

### 3.1. Forming Amino Acids from Sugars and HCN

In this subsection, we calculate the free energy changes for the most likely and direct step-by-step pathway of the reaction of the C_2_ sugar glycolaldehyde (and its thiol analog) with HCN and NH_3_ to form an amino acid, essentially following the Strecker mechanism. Our pathway does not include side reactions/products because our previous work using the same protocol mapped the co-oligomerization of the smaller C_1_ sugar formaldehyde with NH_3_ [42] and also mapped all the potential C_2_ species present after HCN addition [10]. The relevant *G*_rel_ values of all reactants, intermediates, products, and transition states are listed in Table 1. Glycolaldehyde **1a** has a higher *G*_rel_ than mercaptoaldehyde **2a**; we have previously calculated the conversion of **1a** to **2a** in the presence of H_2_S and found it thermodynamically favorable with low barriers (<20 kcal/mol) [43].

**Figure 2:**
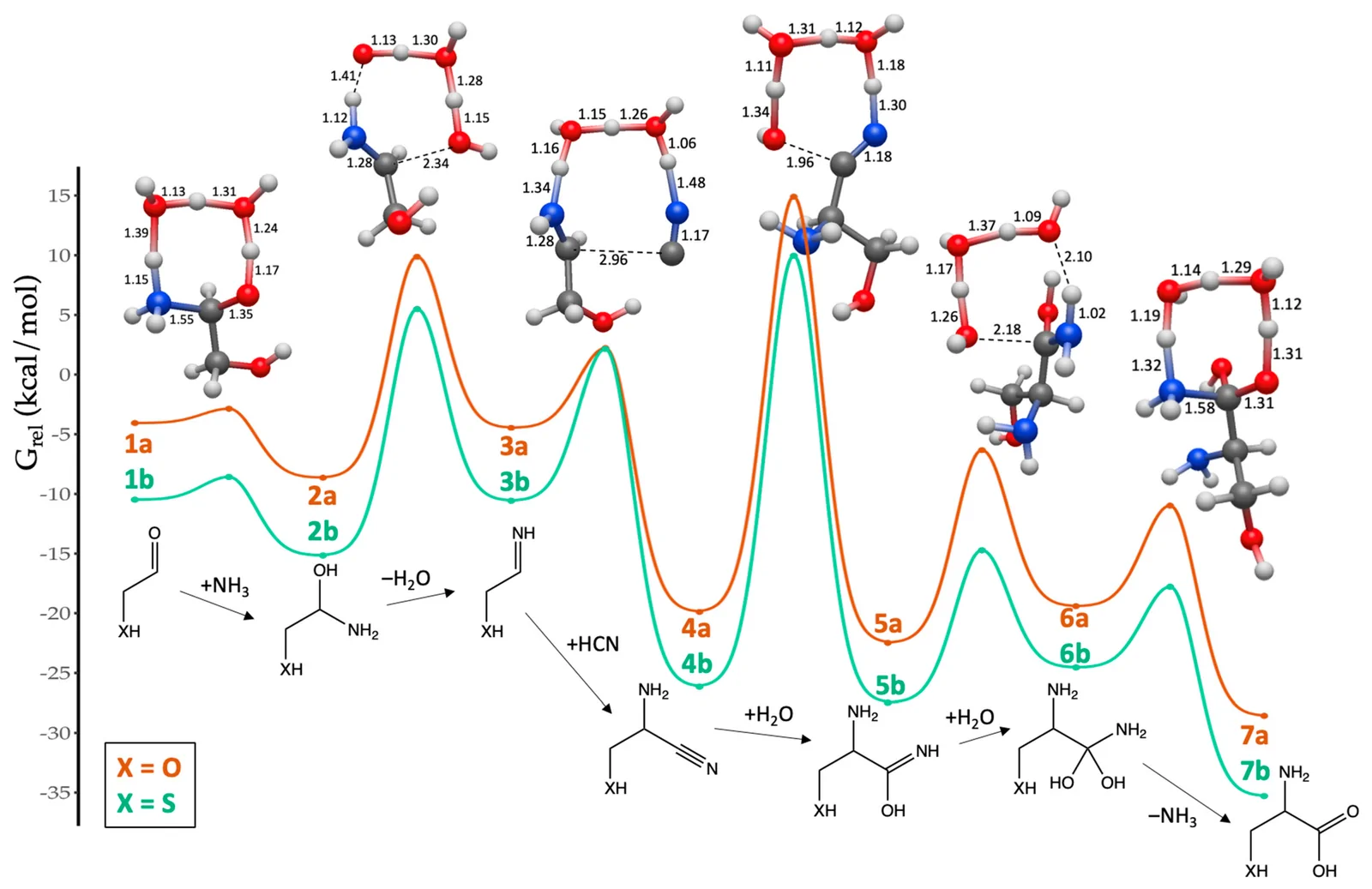
Free energy diagram and transition states for amino acid formation pathways starting from glycolaldehyde (brown) and mercaptoaldehyde (green). Bond distances are in Å.

Figure 2 depicts the energy diagram for the two parallel pathways (glycolaldehyde to serine, mercaptoaldehyde to cysteine). Because HCN has *G*_rel_ = +9.2 kcal/mol, this value must be added to all structures that have not yet incorporated HCN (from **1a**/**1b** to **3a**/**3b**). Thus, the starting point of **1a** + HCN has *G*_rel_ = −13.3 + 9.2 = −4.1 kcal/mol. The transition states shown are for the **1a** to **7a** pathway; the thiol analogs have similar transition states. (XYZ coordinates for all transition states are available in Supplementary Materials).

Starting with **1a**, the first step is the addition of NH_3_ to form **2a**; this reaction is exergonic (∆*G* = −4.6 kcal/mol) with a very low barrier (∆*G*^‡^ = +1.2 kcal/mol). These values are similar to our previous work examining the reactions of CH_2_O and NH_3_ [42]. The optimal transition state includes two additional explicit water molecules to aid proton transfer; in the 8-center transition state the forming and breaking bonds have distances as expected. This is designated **x1a_2a** (the **x** prefix indicates a transition state, and the underscore separates the reactant and product, in this case **1a** and **2a** respectively). The dehydration of **2a** to form the imine **3a** is endergonic (∆*G* = +4.2 kcal/mol) with a modestly low barrier (∆*G*^‡^ = +18.6 kcal/mol). The breaking C…O bond is longer at 2.34 Å but within expectations.

Forming a new C–C bond by adding HCN to **3a** to form the nitrile **4a** is the main thermodynamic driving step; the reaction is significantly exergonic (∆*G* = −15.4 kcal/mol). The optimal transition state, which has a calculated low barrier (∆*G*^‡^ = +7.7 kcal/mol) utilizes the neutral nitrile isomer CNH since this facilitates nitrile having a nucleophilic carbon. Experimentally we expect the cyanide anion to be the reactive species, but our protocol does not perform well with anionic species (see Section 2). With a formal negative charge on carbon, CNH more closely resembles cyanide, but we acknowledge that our calculated barrier may be artificially low by using an “activated” reactant. The forming C…C bond is long at 2.96 Å but gives the correct expected transition state. Our attempts to force neutral HCN with a non-nucleophilic carbon to react constantly led to wrong transition states with C–O bond formation instead of C–C.

Hydration of the nitrile **4a** to the iminol **5a** is slightly exergonic (∆*G* = −2.6 kcal/mol) and also the rate-determining step with a high barrier (∆*G*^‡^ = +34.8 kcal/mol), very similar to our prior work on HCN hydration and known experimental results [27]. Further hydration of the iminol **5a** to the tetrahedral intermediate **6a**, and elimination of NH_3_ leads to the amino acid serine **7a**. The overall reaction of **5a** to **7a** has ∆*G* = −6.1 kcal/mol and barrier ∆*G*^‡^ = +16.2 kcal/mol. In Figure 2, we omitted the iminol as it readily tautomerizes to its thermodynamically more stable amide. As shown in our previous work [10,27], this simply increases the hydration barrier by starting from the more stable amide. Our previous work also found that our protocol significantly underestimates this one particular tautomerization barrier. Our calculations of the amide and these issues are discussed in the Supplementary Materials (which include utilizing a different functional and basis set).

The overall reaction from **1a** to **7a** is exergonic with ∆*G* = −24.0 kcal/mol. Starting from the thiol analog **1b** to form the amino acid cysteine **7b** is similarly exergonic (∆*G* = −24.8 kcal/mol) and the reaction energetics are similar for each step. The only noteworthy difference is the nitrile addition in the sulfur analog (**3b** + HCN to form **4b**) has a barrier ~5 kcal/mol higher (∆*G*^‡^ = +12.7 kcal/mol) than its oxygen analog. There is little difference in the transition states and only small variations in the relevant bond breaking/forming distances for all six steps.

While our previous work on glycine formation provided a free energy map for the plethora of potential C_2_ compounds formed from glycolonitrile [10], it did not cover the crucial C–C bond formation between aldehyde and nitrile. The present study remedies this by finding that this reaction is favorable both thermodynamically and kinetically. In comparison to the energetics for the prebiotic synthesis of glycine (overall ∆*G* is −32 kcal/mol), we find similar results for both serine and cysteine although less exergonic.

### 3.2. Forming Azoles from Sugars and Cyanamide

In this subsection, we calculate the free energy changes for the most likely step-by-step pathway of the reaction of the C_2_ sugar glycolaldehyde (and its thiol analog) with NH_2_CN (cyanamide) to form an azole. The relevant *G*_rel_ values are shown in Table 2.

**Figure 3:**
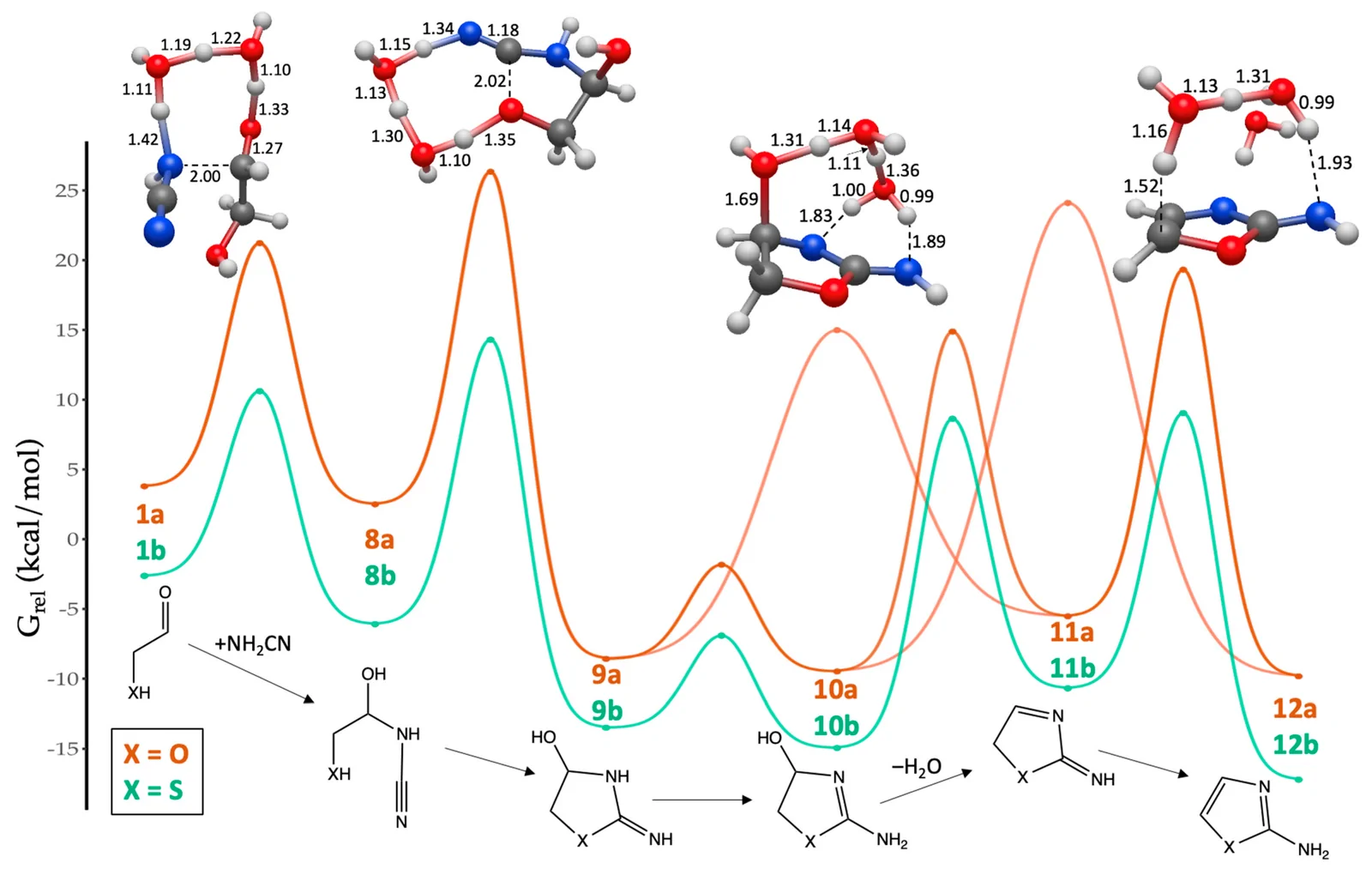
Free energy diagram and transition states for azole formation pathways starting from glycolaldehyde (brown) and mercaptoaldehyde (green). The two lighter brown curved peaks connecting **9a** to **11a** and **10a** to **12a** are alternate paths along the main brown curve. Bond distances are in Å.

Figure 3 depicts the energy diagram for the most favorable pathway when reacting glycolaldehyde with NH_2_CN to form oxazole (in parallel mercaptoaldehyde forms thiazole). Because NH_2_CN has *G*_rel_ = +17.1 kcal/mol, this value must be added to the initial reactants **1a** and **1b**. Thus, the starting point of **1a** + NH_2_CN has *G*_rel_ = −13.3 + 17.1 = +3.8 kcal/mol. The transition states shown are for oxazole formation (the thiol analogs have similar transition states).

For oxazole formation, the first step of adding NH_2_CN to **1a** to form the nitrile **8a** is marginally exergonic (∆*G* = −1.3 kcal/mol) with a modestly low barrier (∆*G*^‡^ = +17.4 kcal/mol). Because of steric hindrance, the forming C…N bond of 2.00 Å in the transition state is longer than for the addition of NH_3_. Other bond distances are as expected. Ring closure of **8a** to **9a** is significantly more exergonic (∆*G* = −11.1 kcal/mol) but has a higher barrier (∆*G*^‡^ = +23.8 kcal/mol). The forming C…O bond is 2.02 Å and this transition state also has the highest overall *G*_rel_ value in the pathway.

Following the main brown curve, tautomerization of **9a** to **10a** is marginally exergonic (∆*G* = −0.9 kcal/mol) with a low barrier (∆*G*^‡^ = +6.8 kcal/mol). Conversely, dehydration to the diamine **11a** is slightly endergonic (∆*G* = +4.0 kcal/mol); however, in the optimized transition state we see that tautomerization and dehydration proceed together, i.e., the transition state **x10a_11a** shifted the water network such that the C…O bond is starting to break (1.69 Å) with water protons almost equidistant between the two nitrogens. Subsequent tautomerization of **11a** to **12a** is slightly exergonic (∆*G* = −4.3 kcal/mol) with a moderate barrier of +24.8 kcal/mol. In the optimal transition state, one water molecule does not participate in bond forming/breaking and is at hydrogen bonding distance to the complex. The C…H bond breaks (1.52 Å) before a proton can be shuttled to the nitrogen at the other end.

Two alternative pathways shown as the light brown curve peaks are shown in Figure 3. Given our observation that dehydration and tautomerization may be concerted, we optimized a transition state directly from **9a** to **11a** which resulted in a similar structure to **x10a_11a** with similar *G*_rel_. Thus, the barrier for this process starting from **9a** is a modest +23.5 kcal/mol. We similarly attempted to bypass **11a** by optimizing a transition state connecting **10a** directly to **12a** but this has a much higher calculated barrier of ~35 kcal/mol (transition state not shown, but XYZ coordinates are provided in Supplementary Materials).

The compounds in Figure 3 are a subset of prebiotic reactions proposed by Ritson et al. [44] and this work allows us to evaluate which pathways are favorable. Their article does not include thermodynamic or kinetic data but our favored pathway is consistent with what they do observe: initial addition of NH_2_CN and the existence of the hydrate **10a** in equilibrium with the oxazole product **12a**. The reaction is relatively slow at pH 7 and 60 °C, consistent with our rate-determining barriers of 24–25 kcal/mol. The possibility of dehydrating **8a** to form an iminonitrile is also mentioned, but we find this reaction to be both thermodynamically and kinetically unfavorable (see Supplementary Materials).

The overall reaction from **1a** + NH_2_CN to **12a** is exergonic (∆*G* = −13.6 kcal/mol) from our calculations. The analogous reaction starting from **1b** to form the thiazole **12b** is overall exergonic by a similar amount (∆*G* = −14.6 kcal/mol). The overall profile for the analogous lowest energy pathway in Figure 3 for the two curves is similar, but there are some differences. The first step converting **1b** to **8b** is more exergonic (∆*G* = −3.5 kcal/mol) while the second step is correspondingly less exergonic compared to its oxygen analog. Three of the barriers are lower in the sulfur analog by 3–5 kcal/mol: the first step **1b** to **8b** (∆*G*^‡^ = +13.2 kcal/mol), ring closure **8b** to **9b** (∆*G*^‡^ = +20.4 kcal/mol) and the final tautomerization of **11b** to **12b** (∆*G*^‡^ = +19.7 kcal/mol), so we might expect thiazole formation to be kinetically more facile than its oxazole counterpart.

In a messy formose mixture, one expects the C_3_ sugar (glyceraldehyde) to also be present. The energetics of this analogous reaction to form an analogous oxazole is not a focus of this article, and the results are shown in the Supplementary Materials. We briefly mention it here because the next subsection discusses a messier system that includes both the C_2_ and C_3_ sugars.

### 3.3. Sugar–Azole Reactions

**Figure 4:**
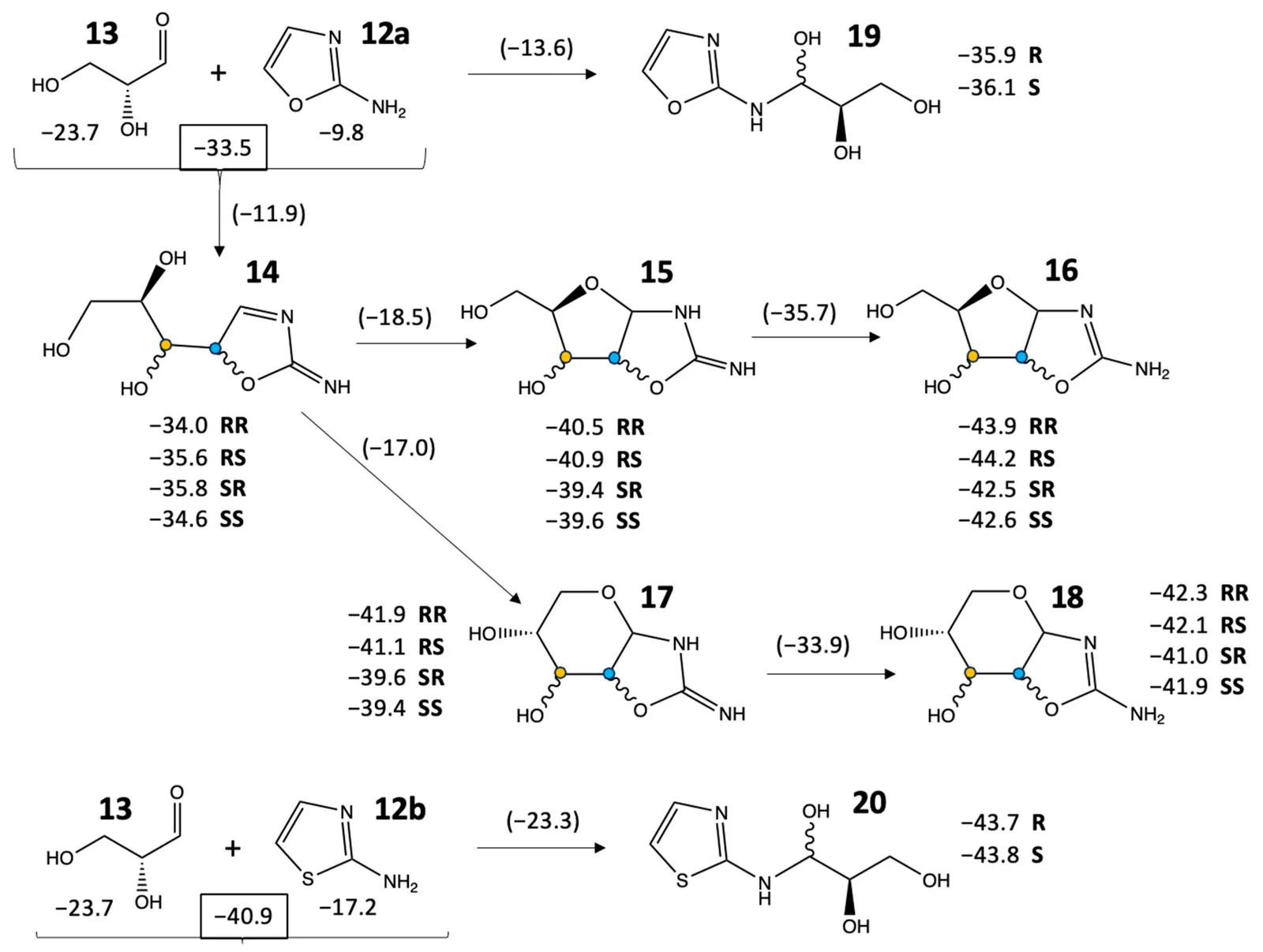
Reaction pathways of D-glyceraldehyde and oxazole (*G*_rel_ values in kcal/mol). Blue dot marks the first stereocenter; orange dot marks the second stereocenter.

We begin this subsection with the reaction of glyceraldehyde and oxazole to form an aminooxazoline that is a precursor to an efficient prebiotic synthesis of pyrimidine ribonucleotides. Our results are shown in Figure 4. Because this strand examines competing reactions, we omit showing messy potential energy curves. Instead, we provide *G*_rel_ values next to each structure and arrow. By comparing the G_rel_ values, one can quickly determine the ∆*G* and ∆*G*^‡^ values for any chemical reaction. Only D-glyceraldehyde **13** is considered, fixing one of the stereocenters. Diastereomer *G*_rel_ values are shown for intermediates and products, while for transition states only the *G*_rel_ value leading to the most stable diastereomeric product is indicated. In addition, the two hydrogens on the carbon bridge in **15**–**18** are always on the same face reducing the number of possible structures.

**Figure 5:**
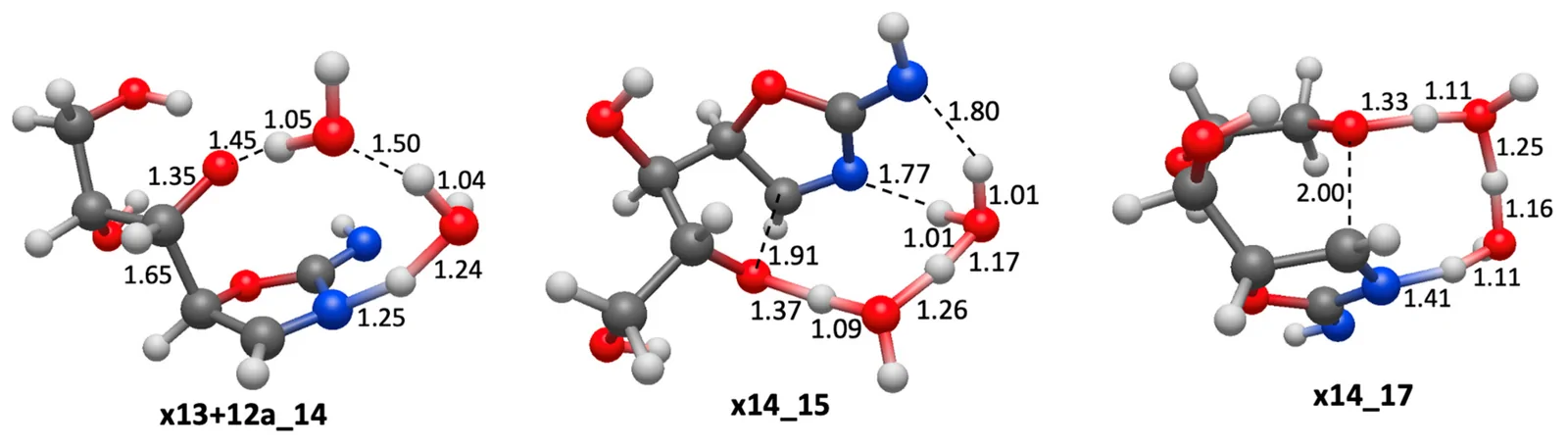
Selected transition states for the addition reaction of D-glyceraldehyde and oxazole (bond distances in Å).

At the starting point of the reaction, the reactants **12a** and **13** have a combined *G*_rel_ of −33.5 kcal/mol. The addition reaction results in **14** and the creation of two new stereocenters. In discussing the ∆*G* and ∆*G*^‡^ values, we will follow the path of the **RS** diastereomer since it leads to the thermodynamically most stable product. The first letter indicates the stereocenter marked by the blue dot, the second letter indicates the stereocenter marked by the orange dot. The formation of **14** is marginally exergonic (∆*G* = −2.1 kcal/mol for **RS**). The other three diastereomers are close in energy, thus we expect all four to be viable intermediates. (For this first step, **SR** is marginally more stable by 0.2 kcal/mol than **RS**, but this difference is within the computational error.) The addition reaction has a modest barrier (∆*G*^‡^ = +21.6 kcal/mol). In the transition state (**x13 + 12a_14**) shown on the left in Figure 5, the forming C…C bond is 1.65 Å. One limitation in our calculation is that to avoid a zwitterionic intermediate, we used an enamine tautomer of **12a** (see Supplementary Materials) whereby the proton hop between the nitrogens has already taken place.

Ring closure of **14** to **15** is more exergonic (∆*G* = −5.3 kcal/mol for **RS**); the four diastereomers are close in energy and although we calculate **RS** to be the most stable, we expect a mixture of all four diastereomers of **15**. This ring closure reaction has a lower barrier (∆*G*^‡^ = +17.1 kcal/mol) than the previous step. In the transition state (**x14_15**) shown in the middle of Figure 5, the forming C…O bond is 1.91 Å and the water network positions one molecule with its protons almost equidistant to the two nitrogens, thus providing a possible direct route to **16**. The tautomerization of **15** to **16** is exergonic (∆*G* = −3.3 kcal/mol) with a low barrier (∆*G*^‡^ = +5.2 kcal/mol). Of the four diastereomers, **RS** is the most stable but with the small spread in energy, all four products are expected.

Our results are in good agreement with the prior experimental work by Anatasi et al. [16]. Starting with a 1:1 ratio of the two reactants, under mild conditions, running this reaction at 40 °C for 12 h leads to the four diastereomeric products (**16**) in over 90% yield. The ratio of diastereomers was reported to be 44:30:13:8, or a spread of slightly over 1 kcal/mol between the most stable and least stable diastereomer. Our calculated spread is 1.7 kcal/mol, comparable to their results (even without concentration corrections) and within the computational errors of our protocol. Our calculated rate-determining step, the addition reaction with its modestly low barrier seems reasonable given their reaction conditions.

There are two side reactions to consider. Instead of forming the five-membered (furanose-like) ring, the ring closure reaction of **14** could lead to a six-membered (pyranose-like) ring. We calculate the reaction of **14** to **17** to also be exergonic (∆*G* = −5.5 kcal/mol for **RS**), similar to the reaction of **14** to **15**. (We choose to follow the **RS** diastereomer for easier comparison with the previous results.) The barrier of +18.6 kcal/mol is marginally higher and in the transition state (**x14_17**) shown on the right in Figure 5, the forming C…O bond has a distance of 2.00 Å. Tautomerization of **17** to **18** is marginally exergonic (∆*G* = −1.0 kcal/mol) with a low barrier of +7.2 kcal/mol. While the four diastereomers of **18** are also very close in energy to each other, the pyranose **18** is on average less stable than the furanose **16** by ~2 kcal/mol, thus one might expect at equilibrium the ratio would be ~10:1 of **16**:**18**, in line with the experimental yield [16]. The overall exergonicity from reactants to the final product **16** is ∆*G* = −9.8 kcal/mol.

The second side reaction is that the amino group of the oxazole could act as a nucleophile adding to the aldehyde of **13** to form **19**. As shown in the top row of Figure 4, this reaction is marginally exergonic (∆*G* = −2.6 kcal/mol for **S**) with a modestly low barrier (∆*G*^‡^ = +19.9 kcal/mol). Since this barrier is ~2 kcal/mol lower than the addition to form **14**, we expect that **19** is readily formed as the kinetic product early in the reaction. However, over time, since **16** is significantly favored thermodynamically, the initial buildup of **19** will reverse and lead to **16** as the dominant product. Experimentally, Islam et al. take advantage of this kinetic sequestration in an elegant series of reactions that allows the separation and selective crystallization of aminooxazalines in a messy mixture containing both the C_2_ and C_3_ sugars [18]. Specifically, they employ the thiazole **12b**. We calculate that the reaction of **13** and **12b** to form **20** (bottom row of Figure 4) is of similar exergonicity (∆*G* = −2.9 kcal/mol for **S**) but with a slightly lower barrier (∆*G*^‡^ = +17.6 kcal/mol) than the oxazole. Our protocol cannot account for the effects of selective crystallization since our calculations assume all substances are in a dilute aqueous solution.

**Figure 6:**
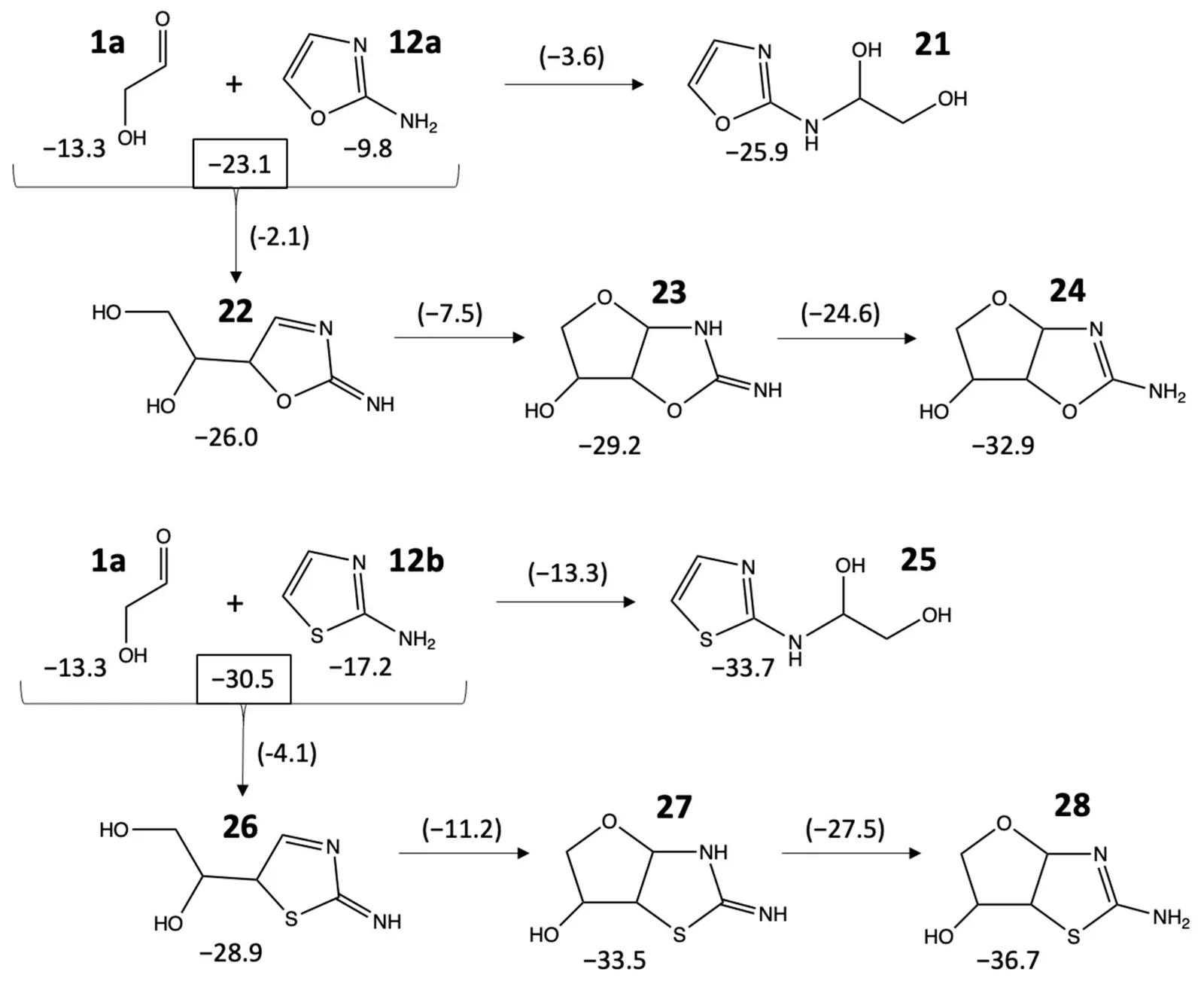
Reaction pathways of glycolaldehyde and oxazole/thiazole (*G*_rel_ values in kcal/mol).

Assuming that C_2_ (glycolaldehyde) is also present in the mixture, we can calculate the energetics of its reaction with both oxazole and thiazole, as shown in Figure 6. With one less stereocenter, and for reading ease and consistency, we only present the energetics for the **RS** diastereomers of **22**–**24** and **26**–**28** (see Supplementary Materials for the free energies of the other diastereomers, which do not change the story). Compounds **21** and **25** only have one stereocenter; we chose to calculate **R** (its enantiomer is degenerate).

The results with glycolaldehyde are very similar to what we found for D-glyceraldehyde. The reaction of **1a** and **12a** to yield **21** is marginally exergonic (∆*G* = −2.9 kcal/mol) with a modest barrier of +19.5 kcal/mol. Alternatively, **1a** and **12a** can add in an aldol-like reaction to form **22**; this reaction is similarly exergonic (∆*G* = −3.0 kcal/mol) with a slightly higher barrier of +21.0 kcal/mol. Ring closure of **22** to form **23** is exergonic (∆*G* = −3.2 kcal/mol) with a barrier of +18.5 kcal/mol. Tautomerization of **23** to **24** is exergonic (∆*G* = −3.7 kcal/mol) with a low barrier of +4.6 kcal/mol.

In the thiazole case, reaction of **1a** and **12b** to form **25** is similarly exergonic (∆*G* = −3.2 kcal/mol) with a slightly lower barrier of 17.2 kcal/mol, similar to what we found for C_3_. However, the aldol-like addition of **1a** and **12b** to form **26** is now marginally endergonic (∆*G* = +1.4 kcal/mol) with a significant barrier of +26.4 kcal/mol. This is consistent with the experimental results under mild conditions [18] where **26**–**28** are not observed and the thiazole is an effective kinetic sequestering agent without complicating the product mixture. If under different experimental conditions **26** can be formed, we calculate its ring closure to **27** to be exergonic (∆*G* = −3.6 kcal/mol) with a modestly low barrier of +17.7 kcal/mol, and its tautomerization to **28** is exergonic (∆*G* = −3.2 kcal/mol) with a low barrier of +6.0 kcal/mol.

Our results in this subsection explore prebiotic reactions leading to precursors of nucleotides with pentose and tetrose sugars from sugars and azoles. They complement known experimental results by providing estimates of the thermodynamic and kinetic parameters influencing which reactions are favored and which are not. In particular, we show that the key C–C bond-forming reaction is aldol-like, complementing the aldol additions of the formose reaction, and that this reaction is the thermodynamic driving force eventually overcoming kinetic sequestration of hemiaminals. But there are many limitations to our calculations since our free energy values are based only on neutral non-zwitterionic molecules (albeit in good agreement with available experimental results), do not account for concentration or crystallization effects, and simply provide baseline values under standard conditions which may not necessarily be the same as prebiotic or experimental conditions.

### 3.4. Coupling of Dehydrated Glyceraldehyde and Cysteine to Form LacCys

**Figure 7:**
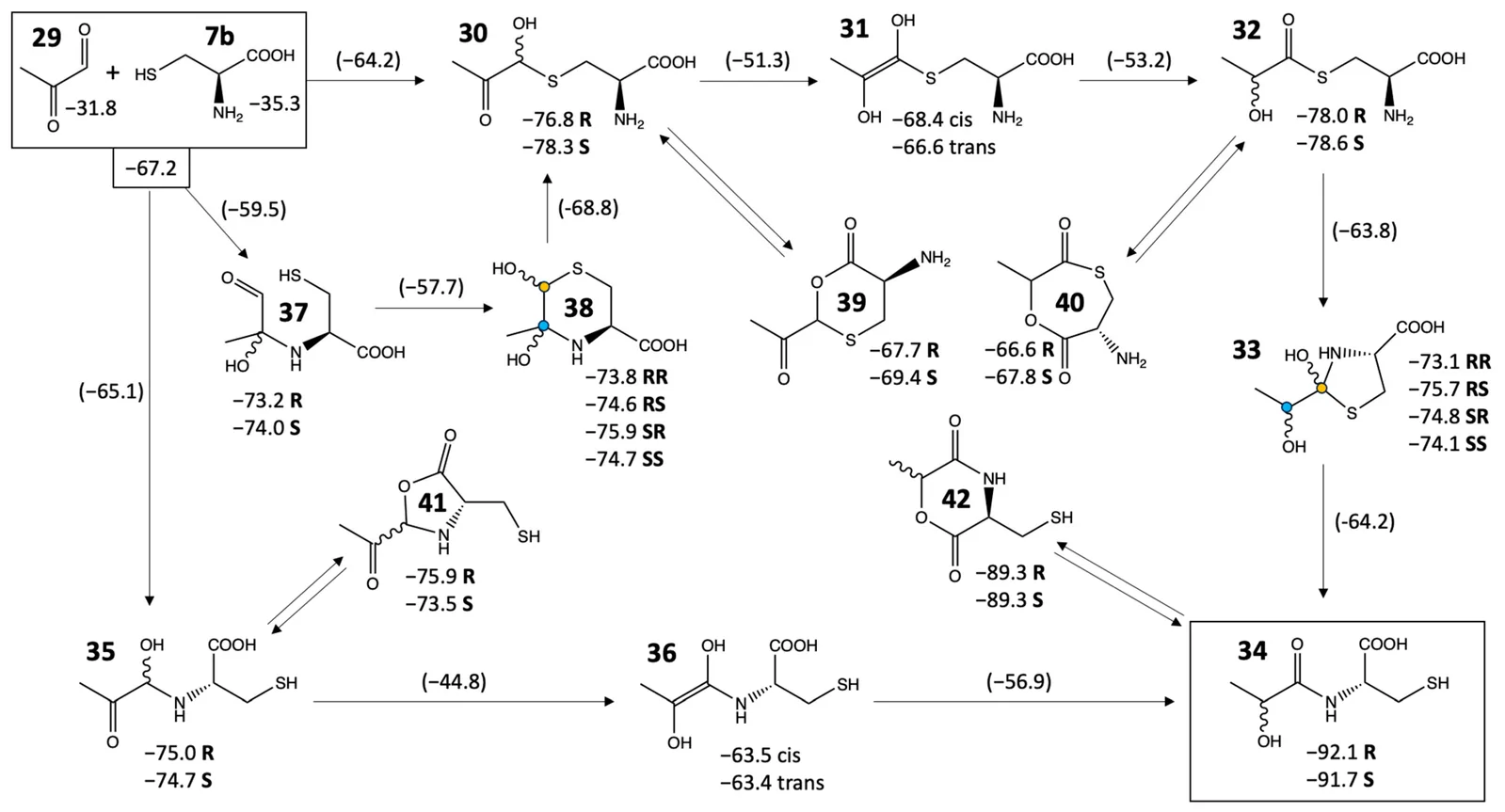
Free energy map for the reaction of methylglyoxal and L-cysteine (*G*_rel_ in kcal/mol). Blue dot marks the first stereocenter; orange dot marks the second stereocenter.

The fourth and final strand we explore is the reaction between a (dehydrated) sugar and an amino acid. We specifically chose the reaction of methylglyoxal and L-cysteine to form LacCys because there is recent experimental evidence for comparison [20], and the proposed mechanism is in concert with (and extends) our previous study on the reaction of sugars and thiols [21]. The reactants, shown in the upper left corner of Figure 7, have a combined *G*_rel_ of −67.2 kcal/mol. Methylglyoxal is the dehydrate of glyceraldehyde.

The experimental paper [20] looks at a range of metabolite adducts produced from reactions with methylglyoxal to probe biochemical pathways towards advanced glycation end-products. Of these, lactoylated amino acids are very abundant, and within this subset, the LacCys adduct is the most abundant. The paper includes experimental data for the aqueous reaction of 10 μM methylglyoxal and 150 μM L-cysteine at pH 7.4 and 37 °C (in the absence of enzymes and cofactors). After 24 h, D-LacCys and L-LacCys are produced in a 1:2 ratio; the yield is 10–15%. They propose a hemithioacetal (HTA) and a thioester as intermediates and estimate a rate constant of 10^−3^ to 10^−4^ s^−1^ for the reaction. They also cite thermodynamic data for the hydration of methylglyoxal prior to reacting with L-cysteine. In a previous work, our protocol showed good agreement with experimental data for methylglyoxal hydration [26].

Our calculated free energy map of this reaction supports HTA being a transient intermediate en route to the formation of a thioester before rearrangement to an amide in the final LacCys product. The most favorable pathway is along the top row of Figure 7, then down the right edge, ending with LacCys in the bottom right corner. As we did in the previous subsection, we show *G*_rel_ values of the diastereomers for all intermediates and products as a reminder that a mixture of products is expected from the narrow range of values. For ease of reading, only the transition state with the lowest *G*_rel_ is shown in Figure 7 (with the range of values tabulated in Supplementary Materials for all diastereomers). When we quote ∆*G* values, we will provide an average value with no decimal place, with the understanding that there are small differences for specific pathways that do not change the overall story.

From our calculations, the reaction of **29** and **7b** to form the HTA **30** is quite exergonic (∆*G* = −10 kcal/mol) with a low barrier of +3 kcal/mol. In this reaction, the thiol acts as the nucleophile attacking the aldehyde carbon. To form the thioester, the limits of our protocol [21] require us to split this reaction into two steps: first the enolization of **30** to **31**, followed by an enol-keto shift to form the thioester **32**. The overall reaction of **30** to **32** is marginally exergonic (∆*G* = −1 kcal/mol) with a modestly high barrier of ~26 kcal/mol. (The enol **31** is a transient intermediate.) Ring closure from **32** to **33** via the amino group as the nucleophile attacking the thioester is slightly endergonic (∆*G* = +4 kcal/mol) with a relatively low barrier of +12 kcal/mol. Ring opening of **33** to the final product **34** is significantly exergonic (∆*G* = −18 kcal/mol) with a low barrier of +10 kcal/mol. Hence, while the thioester **32** may be trapped as an intermediate, **33** is likely to be transient. Our calculated final product **34** has a 0.4 kcal/mol difference between the two diastereomers, or roughly a 2:1 ratio of **R** to **S**; however, this magnitude is within the computational error so we do not attach too much significance to our calculated ratio. Without knowing the Arrhenius pre-exponential factor, we cannot directly compare our calculated barriers to the experimental rate constant, but a crude approximation suggests we are within an order of magnitude to reported experimental results (see Supplementary Materials).

**Figure 8:**
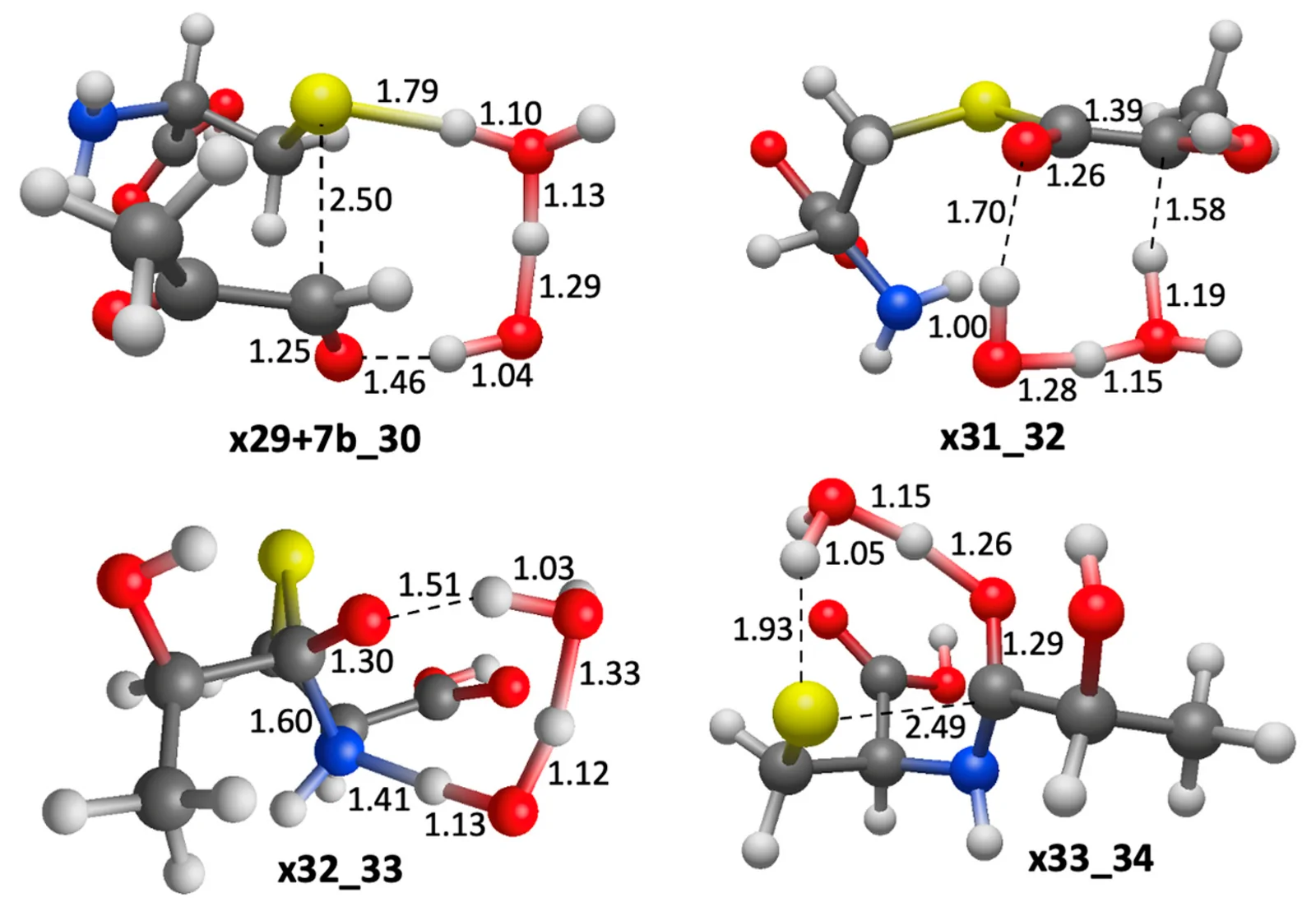
Selected transition states along the pathway for the reaction of methylglyoxal and L-cysteine to form the LacCys adduct (bond distances in Å).

Four of the transition states along this pathway are shown in Figure 8. For the initial addition of **29** and **7b** to form **30**, the forming C…S bond is 2.50 Å and the breaking S…H bond is 1.79 Å (structure **x29 + 7b_30**). For the formation of thioester **32** from the enol **31** (structure **x31_32**), the O…H bond is mostly broken at 1.70 Å, even though the forming C…H bond is still relatively long at 1.58 Å. There are no surprises for the transition state of ring closure (structure **x32_33**); the forming C…N bond is 1.60 Å and the breaking N…H bond is 1.41 Å, while the H…O bond at 1.51 Å is just starting to form. Ring opening of **33** to **34** has a forming C…S bond of 2.49 Å (structure **x33_34**) as expected, while the breaking S…H bond is on the longer side at 1.93 Å but still within a reasonable range. Only one explicit bridging water molecule (unlike two in the other cases) was needed for the optimal structure.

The 10–15% yield of LacCys under experimental conditions suggests the possible formation of other intermediates and side products (not identified experimentally). A competing reaction for the very first step is for the amino group to act as the nucleophile attacking the aldehyde carbon. This reaction converting **29** and **7b** into **35** is exergonic (∆*G* = −8 kcal/mol) although less so than forming **30**. It also has a very low barrier of +2 kcal/mol, not too different than forming **30**. An analogous two-step reaction, via the transient enol intermediate **36** (given our protocol), converts **35** to LacCys **34**. While the overall reaction is quite exergonic, the barrier is high at +30 kcal/mol, so this pathway to LacCys is rather slow and unlikely to proceed significantly under experimental conditions.

Another competing reaction for the first step is that the amino group of **7b** could attack the ketone of **29** to form **37**. While this reaction is exergonic (∆*G* = −6 kcal/mol), it is less so than the previously discussed reactions of **29** and **7b**. The calculated barrier is also higher at +8 kcal/mol although still a low number and therefore kinetically very feasible. The most likely subsequent reaction is an intramolecular attack of the thiol on the aldehyde carbon in **37** to form **38**, which is mildly exergonic (∆*G* = −2 kcal/mol) with a modestly low barrier of +18 kcal/mol. Ring opening of **38** to form **30** is also mildly exergonic (∆*G* = −2 kcal/mol) with a low barrier of +6 kcal/mol. Thus, we do not expect significant accumulation of **37** or **38** since they will eventually form **30**.

The presence of a carboxylic acid and an alcohol allow for several lactones to be formed. Four examples are shown in Figure 7. The conversion of **30** to **39** is significantly endergonic (∆*G* = +11 kcal/mol) and so is the conversion of **32** to the 7-membered ring **40** (also with ∆*G* = +11 kcal/mol). Both these lactones are unlikely to accumulate as side products. On the other hand, the conversion of **35** to the 5-membered ring **41** is thermodynamically even, and we expect the formation of **35** and **41** as side products to reduce the yield of LacCys. While LacCys **34** can also be sequestered as lactone **42**, we calculate this reaction to be +3 kcal/mol endergonic. There are other side reactions not considered in this work including the self-oligomerization of methylglyoxal [26] or the reaction of either reactant with any of the intermediates shown. These could reduce the overall yield, in addition to the rate-determining barrier of +26 kcal/mol which kinetically hinders thioester formation.

In summary, we find that the most favorable pathway for formation of LacCys proceeds via HTA and thioester intermediates; however, there are side reactions that may lead to other products reducing the final yield of LacCys. Our calculated values show some correspondence to experimental results but a direct comparison is not straightforward (see Supplementary Materials); our values may be of the correct magnitude and seemingly close matches might be coincidental or within the experimental or computational error bars. While the referenced experimental work was not exploring prebiotic chemistry, our interest in the role of thioesters in origins-of-life scenarios led us to consider that feasible reactions between cysteine and aldehydes may have later led to the evolution of enzymes to control and facilitate such reactions.

## 4. Conclusions

In this article, we presented a computational study of four connected strands, a small subset in a larger network of potential prebiotic reactions. The first strand explored C–C bond formation between sugars and HCN to form amino acids. Examining the C_2_ sugar (and its thiol analog) extended our previous map of C_1_ reacting with HCN. It is also an alternative way to build new C–C bonds beyond our previous free energy maps on formose aldol reactions. The second strand explored the same C_2_ sugar reacting with NH_2_CN to form azoles. These further condense with C_2_ and C_3_ sugars to form aminooxazoles with aldol-like C–C bond formation in the third strand, where we also considered competing reactions that shed light on the relative thermodynamics and kinetics governing a complex mixture. Our fourth strand considered the coupling of one of the amino acids from the first strand, and the (dehydrated) C_3_ sugar from the third strand, to form a metabolite via a thioester intermediate, extending our previous maps of such chemistry.

Like other computational studies, our results are limited by our protocol. We only calculated neutral compounds, applied a simple entropy correction factor, used an implicit solvent (and a couple of explicit water molecules in transition states), and our density functional and basis set are modest. Error cancelation is a hallmark of quantum chemistry, and our protocol performs reasonably well when we are able to compare to available experimental results under standard conditions, not an easy task when dealing with complex mixtures with a variety of experimental protocols. Our hope is that the present work provides more complementary information to experimentalists working on prebiotic systems. This work complements our previous work, increasing the coverage of baseline thermodynamic and kinetic information and extending our free energy map, and we hope this helps prune some of the pathways as we constrain chemical scenarios that lead to life’s origins.

## Figures and Tables

**Table 1:** *G*_rel_ values for amino acid formation pathways.

Compound	*G*_rel_ (kcal/mol)	Compound	*G*_rel_ (kcal/mol)
**1a**	−13.3	**1b**	−19.7
**2a**	−17.9	**2b**	−24.4
**3a**	−13.7	**3b**	−19.8
**4a**	−19.9	**4b**	−26.2
**5a**	−22.5	**5b**	−27.5
**6a**	−19.4	**6b**	−24.5
**7a**	−28.6	**7b**	−35.3
**x1a_2a**	−12.1	**x1b_2b**	−17.8
**x2a_3a**	+0.7	**x2b_3b**	−3.7
**x3a_4a**	+2.2	**x3b_4b**	+2.1
**x4a_5a**	+14.9	**x4b_5b**	+10.0
**x5a_6a**	−6.3	**x5b_6b**	−14.7
**x6a_7a**	−11.0	**x6b_7b**	−17.8

**Table 2:** *G*_rel_ values for azole formation pathways.

Compound	*G*_rel_ (kcal/mol)	Compound	*G*_rel_ (kcal/mol)
**8a**	+2.5	**8b**	−6.1
**9a**	−8.6	**9b**	−13.5
**10a**	−9.5	**10b**	−14.9
**11a**	−5.5	**11b**	−10.7
**12a**	−9.8	**12b**	−17.2
**x1a_8a**	+21.2	**x1b_8b**	+10.6
**x8a_9a**	+26.3	**x8b_9b**	+14.3
**x9a_10a**	−1.8	**x9b_10b**	−6.9
**x10a_11a**	+14.9	**x10b_11b**	+8.6
**x11a_12a**	+19.3	**x11b_12b**	+9.0

## Data Availability

The data presented in this study are available in Supplementary Materials.

## Supplementary Materials

The following supporting information can be downloaded at: https://www.mdpi.com/article/10.3390/life16091531/s1, Table S1: Energy breakdown of molecules and transition states; Table S2: Free energy comparison between the two methods; Table S3: *G*_rel_ values for all transition state diastereomers; Figure S1: *G*_rel_ values for conversion of iminol to amide followed by hydration to the tetrahedral intermediate; Figure S2: Energetics for the iminonitrile dehydration; Figure S3: Enamine tautomer of oxazole/thiazole; Figure S4: Free energies for the reaction of glyceraldehyde and cyanamide to form its corresponding oxazole; Figure S5: Free energies for the reaction of glycolaldehyde and oxazole.
