# Supplementary material for: Strands in a Web: Free Energies of Select Prebiotic Reactions with Sugars, Nitriles and Azoles
Source: Life (Basel). 2026 Sep 15;16(9):1531. doi: 10.3390/life16091531 (PMC13608698; doi:10.3390/life16091531)
Supplement: Supplementary file 1 [file life-16-01531-s001.zip › life-4545595-supplementary.pdf]

Supporting Information for:

Strands in a Web: Selected Prebiotic Reactions of Sugars, Nitriles and Azoles

By Jeremy Kua* and Alexandra N. Mateescu
University of San Diego, 5998 Alcala Park, San Diego, CA 92110, U.S.A.
*jkua@sandiego.edu

Part 1: Energy Breakdown for all molecules in this study

Table S1 contains the energy breakdown for calculated structures in this article. The computational methods section in the main text describes each of these categories: E_{elec} is the electronic energy in atomic units. All other values are in kcal/mol. E_{solv} is the solvation energy when the molecule is embedded in a dielectric. H_{corr} includes the zero-point-energy and standard enthalpy corrections to 298 K. The entropic correction ($-0.5TS_{corr}$) is half of the standard gas-phase entropy correction at 298 K. The last column (G_{298}) is the total free energy obtained by adding the other four columns (where the atomic units of E_{elec} are converted to kcal/mol by multiplying 627.5096. The transition states are labeled with a double-headed arrow between each structure. The negative frequency eigenvalue (in cm^{-1}) for each transition state is included in the table and the corresponding eigenvectors do correspond to the intrinsic reaction coordinate for the desired reaction.

Table S1. Energy breakdown of molecules and transition states. Units are kcal/mol unless indicated otherwise in parentheses.

	E_{elec} (a.u.)	E_{solv}	H_{corr}	$-0.5TS_{corr}$	G_{298}	G_{rel}
**Reference molecules**						
H_2	-1.17957	1.62	8.39	-4.64	-734.82	0.0
CO_2	-188.64132	2.10	9.59	-7.61	-118370.16	0.0
H_2O	-76.44745	-9.75	15.74	-6.92	-47972.44	0.0
NH_3	-56.57607	-4.08	23.89	-7.17	-35489.39	0.0
H_2S	-399.42206	-2.09	11.92	-7.53	-250638.88	0.0
**All other structures**						
HCN	-93.45216	-3.49	12.51	-7.16	-58640.27	9.2
NH_2CN	-148.83014	-10.02	24.20	-8.82	-93386.99	17.1
**1a**	-229.11323	-8.73	41.55	-10.06	-143748.00	-13.3
**2a**	-285.70713	-15.86	68.98	-11.12	-179241.96	-17.9
**3a**	-209.22953	-11.67	49.97	-10.10	-131265.34	-13.7
**4a**	-302.71656	-16.83	65.34	-12.00	-189921.04	-19.9
**5a**	-379.20360	-14.16	84.27	-12.29	-237896.07	-22.5
amide **a**	-379.21953	-18.18	84.04	-12.86	-237910.90	-37.3
**6a**	-455.67012	-17.50	102.71	-13.28	-285865.44	-19.4
**7a**	-399.09288	-14.34	76.32	-12.58	-250385.21	-28.6

**1b**	-552.09154	-6.01	38.61	-10.71	-346420.85	-19.7
**2b**	-608.68534	-13.32	65.92	-11.62	-381914.92	-24.4
**3b**	-532.20945	-7.14	46.54	-10.75	-333937.89	-19.8
**4b**	-625.69348	-14.77	62.17	-12.47	-392593.74	-26.2
**5b**	-702.17656	-12.88	80.94	-13.03	-440567.50	-27.5
amide **b**	-702.19885	-15.21	80.85	-13.24	-440584.12	-44.1
**6b**	-778.63971	-18.4	99.34	-14.05	-488537.01	-24.5
**7b**	-722.06898	-12.86	72.93	-13.22	-453058.36	-35.3
**8a**	-377.96227	-17.50	68.77	-12.59	-237136.27	2.5
**9a**	-377.98131	-17.99	69.30	-11.76	-237147.35	-8.6
**10a**	-377.98602	-15.89	69.36	-11.86	-237148.24	-9.5
**11a**	-301.50212	-16.17	50.33	-10.54	-189171.85	-5.5
**12a**	-301.52048	-9.42	50.84	-10.58	-189176.15	-9.8
**8b**	-700.93603	-19.47	65.42	-13.15	-439811.29	-6.1
**9b**	-700.95715	-16.93	67.60	-12.02	-439818.69	-13.5
**10b**	-700.96193	-15.11	67.54	-12.24	-439820.15	-14.9
**11b**	-624.48151	-12.68	48.44	-11.06	-391843.45	-10.7
**12b**	-624.49895	-8.81	48.97	-11.04	-391849.97	-17.2
**13**	-343.67347	-19.21	63.76	-11.87	-215625.72	-23.6
**14 RR**	-645.21740	-24.14	116.98	-15.14	-404802.41	-34.0
**14 RS**	-645.21473	-27.03	116.67	-15.20	-404804.00	-35.6
**14 SR**	-645.21146	-29.17	116.72	-15.35	-404804.19	-35.8
**14 SS**	-645.21607	-25.60	117.00	-15.14	-404803.02	-34.6
**15 RR**	-645.23632	-20.13	117.81	-14.62	-404808.92	-40.5
**15 RS**	-645.23399	-21.91	117.85	-14.72	-404809.30	-40.9
**15 SR**	-645.23366	-21.04	118.1	-14.52	-404807.77	-39.4
**15 SS**	-645.22371	-26.56	117.56	-14.90	-404807.97	-39.6
**16 RR**	-645.23696	-22.9	117.77	-14.74	-404812.25	-43.9
**16 RS**	-645.23489	-24.79	118.06	-14.74	-404812.56	-44.2
**16 SR**	-645.23170	-25.5	118.24	-14.56	-404810.90	-42.5
**16 SS**	-645.22789	-27.38	117.95	-14.86	-404810.98	-42.6
**17 RR**	-645.23464	-23.34	118.31	-14.30	-404810.26	-41.9
**17 RS**	-645.22842	-25.97	118.02	-14.51	-404809.48	-41.1
**17 SR**	-645.23105	-23.12	118.27	-14.47	-404808.00	-39.6
**17 SS**	-645.23277	-21.93	118.31	-14.38	-404807.76	-39.4
**18 RR**	-645.23430	-24.32	118.64	-14.29	-404810.69	-42.3
**18 RS**	-645.22548	-28.8	118.13	-14.62	-404810.48	-42.1
**18 SR**	-645.23221	-23.8	118.27	-14.45	-404809.39	-41.0
**18 SS**	-645.22953	-26.13	118.14	-14.56	-404810.27	-41.9
**19 R**	-645.23041	-18.39	117.48	-15.07	-404804.26	-35.9
**19 S**	-645.22774	-19.91	117.39	-15.33	-404804.45	-36.1
**20 R**	-968.21122	-16.78	115.64	-15.55	-607478.53	-43.7
**20 S**	-968.20685	-19.15	115.35	-15.77	-607478.66	-43.8
**21**	-530.66380	-12.29	95.49	-13.53	-332926.96	-25.9
**22**	-530.64333	-24.34	94.74	-13.70	-332927.08	-26.0
**23**	-530.66152	-17.71	95.74	-13.12	-332930.29	-29.2

**24**	-530.66181	-21.31	95.87	-13.13	-332933.95	-32.9	
**25**	-853.64121	-12.57	93.47	-14.00	-535601.15	-33.7	
**26**	-853.61428	-23.45	92.81	-14.36	-535596.16	-28.7	
**27**	-853.62715	-21.78	93.71	-13.66	-535600.97	-33.5	
**28**	-853.63155	-22.17	93.74	-13.78	-535604.20	-36.7	
**29**	-267.23015	-5.36	44.52	-11.11	-167661.44	-31.8	
**30 R**	-989.33454	-17.41	121.47	-16.65	-620729.51	-76.8	
**30 S**	-989.33516	-18.63	121.39	-16.43	-620730.98	-78.3	
**31** (cis)	-989.32015	-16.78	121.70	-18.13	-620721.10	-68.4	
**31** (trans)	-989.31826	-17.57	121.71	-16.72	-620719.28	-66.6	
**32 R**	-989.33216	-19.58	121.33	-17.00	-620730.68	-78.0	
**32 S**	-989.33409	-19.22	121.34	-16.79	-620731.31	-78.6	
**33 RR**	-989.32450	-21.6	122.16	-15.74	-620725.80	-73.1	
**33 RS**	-989.32719	-22.56	122.21	-15.78	-620728.44	-75.7	
**33 SR**	-989.32797	-20.66	121.87	-15.90	-620727.48	-74.8	
**33 SS**	-989.33099	-18.99	122.50	-15.64	-620726.82	-74.1	
**34 R**	-989.35277	-19.69	120.14	-16.88	-620744.79	-92.1	
**34 S**	-989.34878	-21.86	120.27	-16.98	-620744.43	-91.7	
**35 R**	-989.32692	-18.61	119.95	-16.87	-620727.67	-75.0	
**35 S**	-989.32585	-18.77	119.71	-16.92	-620727.45	-74.7	
**36** (cis)	-989.31394	-16.05	120.34	-16.53	-620716.23	-63.5	
**36** (trans)	-989.31048	-17.36	120.08	-17.02	-620716.12	-63.4	
**37 R**	-989.33007	-16.67	121.34	-16.45	-620725.90	-73.2	
**37 S**	-989.32907	-18.01	121.14	-16.35	-620726.71	-74.0	
**38 RR**	-989.32763	-20.47	122.34	-15.76	-620726.48	-73.8	
**38 RS**	-989.32680	-21.73	122.26	-15.77	-620727.31	-74.6	
**38 SR**	-989.32605	-24.21	122.68	-15.45	-620728.57	-75.9	
**38 SS**	-989.33301	-18.70	122.63	-15.39	-620727.42	-74.7	
**39 R**	-912.84637	-15.85	103.24	-15.49	-572747.96	-67.7	
**39 S**	-912.85007	-15.94	103.53	-15.07	-572749.66	-69.4	
**40 R**	-912.84751	-14.88	103.52	-14.92	-572746.86	-66.6	
**40 S**	-912.84940	-14.77	103.5	-15.02	-572748.06	-67.8	
**41 R**	-912.84970	-20.68	102.06	-15.34	-572755.91	-75.6	
**41 S**	-912.85199	-16.82	101.93	-15.51	-572753.79	-73.5	
**42 R**	-912.87297	-20.30	102.45	-15.19	-572769.60	-89.3	
**42 S**	-912.87608	-18.59	102.59	-15.09	-572769.59	-89.3	
**Transition states**							Freq(cm^{-1})
**x1a_2a**	-438.61793	-28.20	97.81	-13.72	-275181.07	-12.1	-1034
**x2a_3a**	-438.60792	-20.01	96.60	-14.25	-275168.34	0.7	-873
**x3a_4a**	-455.60735	-23.42	92.90	-15.30	-285843.81	2.2	-896
**x4a_5a**	-532.06949	-20.64	111.39	-15.60	-333803.57	14.9	-1171
**x5a_6a**	-608.58026	-22.54	131.62	-16.37	-381797.25	-6.3	-827
**x6a_7a**	-608.57299	-31.47	130.94	-15.98	-381801.90	-11.0	-1227
**x1b_2b**	-761.59592	-24.67	94.56	-14.36	-477853.22	-17.8	-1000
**x2b_3b**	-761.57397	-22.98	93.60	-14.79	-477839.15	-3.7	-811
**x3b_4b**	-778.57597	-21.34	91.29	-16.38	-488510.32	2.1	-318

**x4b_5b**	-855.04362	-20.22	109.07	-15.72	-536474.95	10.0	-1073
**x5b_6b**	-931.55412	-23.88	127.55	-16.59	-584472.07	-14.7	-978
**x6b_7b**	-931.55360	-27.97	128.07	-16.41	-584475.13	-17.8	-1157
**x1a_8a**	-530.85737	-25.49	96.53	-15.38	-333062.44	21.2	-1067
**x8a_9a**	-530.86113	-19.64	97.39	-14.61	-333057.31	26.3	-1028
**x9a_10a**	-530.91311	-18.29	99.77	-13.90	-333085.49	-1.8	-864
**x10a_11a**	-530.87992	-21.85	99.39	-14.07	-333068.78	14.9	-704
**x11a_12a**	-530.87244	-22.04	99.74	-14.49	-333064.34	19.3	-1145
**x9a_11a**	-530.87991	-21.91	99.51	-14.03	-333068.67	15.0	-698
**x10a_12a**	-454.38182	-25.58	80.71	-13.29	-285087.12	24.1	-944
**x1b_8b**	-853.84090	-23.47	93.43	-16.08	-535739.48	10.6	-985
**x8b_9b**	-853.83196	-29.57	96.94	-15.41	-535735.79	14.3	-535
**x9b_10b**	-853.88619	-18.81	97.93	-14.34	-535757.00	-6.9	-834
**x10b_11b**	-853.85842	-21.04	98.33	-14.39	-535741.46	8.6	-596
**x11b_12b**	-853.85360	-20.87	96.19	-15.04	-535741.05	9.0	-801
**x13+12a_19**	-798.12091	-24.63	144.32	-18.04	-500726.89	-13.6	-1300
**x13+12a_14**	-798.11875	-27.27	146.66	-17.39	-500725.18	-11.9	-731
**x14_15**	-798.13689	-23.61	147.58	-17.17	-500731.76	-18.5	-668
**x15_16**	-798.15746	-26.32	145.86	-17.05	-500748.97	-35.7	-1503
**x14_17**	-798.12992	-25.97	146.70	-16.92	-500730.38	-17.1	-1046
**x17_18**	-798.14907	-29.92	145.91	-16.93	-500747.15	-33.9	-1052
**x13+12b_20**	-1121.10021	-26.50	142.84	-18.26	-703403.07	-23.3	-1360
**x1a+12a_21**	-683.54977	-21.15	122.16	-16.48	-428849.52	-3.6	-1343
**x1a+12a_22**	-683.54170	-26.62	123.84	-16.22	-428847.98	-2.1	-781
**x22_23**	-683.56327	-21.13	125.69	-15.46	-428853.42	-7.5	-838
**x23_24**	-683.58456	-22.67	123.56	-15.53	-428870.51	-24.6	-1499
**x1a+12b_25**	-1006.52890	-22.50	120.32	-16.89	-631525.61	-13.3	-1412
**x1a+12b_26**	-1006.51199	-26.16	122.65	-17.01	-631516.45	-4.1	-810
**x26_27**	-1006.53783	-18.94	123.63	-16.07	-631523.53	-11.2	-791
**x27_28**	-1006.55218	-24.16	121.60	-16.16	-631539.87	-27.5	-1475
**x29+7b_30**	-1142.22353	-35.42	149.78	-19.96	-716661.83	-64.2	-530
**x30_31**	-1142.21243	-30.33	149.88	-19.20	-716648.91	-51.3	-1226
**x31_32**	-1142.22741	-24.75	151.60	-19.00	-716650.82	-53.2	-976
**x32_33**	-1142.23560	-32.28	152.64	-17.92	-716661.37	-63.8	-683
**x33_34**	-1065.77509	-24.31	135.89	-16.84	-668689.36	-64.2	-765
**x29+7b_35**	-1142.24778	-23.95	151.17	-18.44	-716662.67	-65.1	-778
**x35_36**	-1142.20158	-30.17	149.19	-18.96	-716642.40	-44.8	-1203
**x36_34**	-1142.23430	-23.33	150.66	-18.83	-716654.49	-56.9	-1107
**x29+7b_37**	-1142.23050	-27.42	149.65	-18.67	-716657.05	-59.5	-822
**x37_38**	-1142.21863	-34.16	150.50	-18.52	-716655.34	-57.7	-699
**x38_30**	-1142.23781	-35.27	151.75	-17.64	-716666.35	-68.8	-894

Part 2: Iminol to amide tautomerization

As discussed in Section 3.1 of the main article, the iminol to amide tautomerization is thermodynamically favorable, however the amide sits in a thermodynamic sink. Hence, the subsequent barrier for the reaction leading to amino acid is correspondingly higher. The G_{rel} values for the amide and the two transition states are shown in Figure S1. Converting **5a** and **5b** to their respective amides is significantly exergonic (ΔG = –14.8 and –16.6 kcal/mol respectively) and the barriers are 3.0 and 0.0 kcal/mol respectively. This barrier is artificially low as discussed in the next section. Because the amide is so much more stable than the iminol, the hydration barriers to form the tetrahedral intermediate are increased to ~30 kcal/mol instead of ~16 kcal/mol from the iminol. This difference essentially comes from the exergonicity of the iminol to amide tautomerization.

Figure S1. G_{rel} values (kcal/mol) for conversion of iminol to amide followed by hydration to the tetrahedral intermediate (**a**: X = O, **b**: X = S).

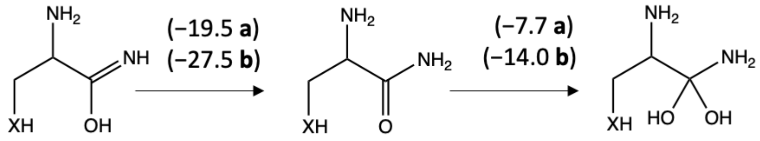

−22.5 **5a**
−27.5 **5b**

−37.3 amide **a**
−44.1 amide **b**

−19.4 **6a**
−24.5 **6b**

Part 3: Energetics for amino acid formation pathways with ωB97M-V/def2-TZVPP

As discussed in Section 3.1 of the main article, our calculated iminol to amide tautomerization barriers are artificially low. We encountered this in our previous study for glycine formation (starting from formaldehyde, HCN and NH_3) [Thrush & Kua, *J. Phys. Chem. A* **2018**, *122*, 6769-6779, also cited in the main article]. In that study we proposed that for this one particular reaction, a suitable entropic scaling factor should be ~0.35 rather than the 0.5 found to be satisfactory for all the other reactions.

An alternative would be to use a more robust and modern density functional theory method and a larger basis set. We chose to use ωB97M-V/def2-TZVPP and our results are shown below. In short, this more expensive method yields similar thermodynamics (as indicated by ΔG values) but increases all the barriers significantly (as indicated by $\Delta G^{\ddagger}$ values) to unrealistically high values, as shown in Table S2. The majority of these reactions are facile at room temperature suggesting barriers below 20 kcal/mol with the exception of nitrile hydration (for HCN the experimental value is ~30 kcal/mol).

Table S2. Free energy comparison between the two methods (values in kcal/mol).

Reaction	B3LYP-D3/6-311G**		ωB97M-V/def2-TZVPP	
X = O	ΔG	$\Delta G^{\ddagger}$	ΔG	$\Delta G^{\ddagger}$
**1a** to **2a**	–4.6	+1.2	–5.0	+10.0
**2a** to **3a**	+4.2	+18.6	+2.8	+30.5
**3a** to **4a**	–15.4	+7.7	–17.3	+17.4
**4a** to **5a**	–2.6	+34.8	+0.9	+50.5
**5a** to amide	–14.8	+3.0	–15.2	+11.2
amide to **6a**	+17.9	+29.6	+18.6	+43.8
**6a** to **7a**	–9.2	+8.4	–8.3	+15.5
X = S	ΔG	$\Delta G^{\ddagger}$	ΔG	$\Delta G^{\ddagger}$
**1b** to **2b**	–4.7	+1.9	–5.3	+10.5
**2b** to **3b**	+4.6	+20.7	+3.7	+31.5
**3b** to **4b**	–15.6	+12.7	–18.1	+21.2
**4b** to **5b**	–1.3	+36.2	+1.9	+51.4
**5b** to amide	–16.6	+0.0	–16.1	+9.5
amide to **6b**	+19.6	+30.1	+19.2	+41.2
**6b** to **7b**	–8.8	+7.4	–10.1	+14.3

Part 4: Additional intermediates in the azole formation pathway

As discussed in Section 3.2 of the main article, the dehydration of **8a** to an iminonitrile is both thermodynamically and kinetically unfavorable. From Figure S2, we see that the reaction is endergonic (ΔG = +3.1 kcal/mol) and the barrier is 24.6 kcal/mol.

Figure S2. Energetics for the iminonitrile dehydration (G_{rel} values in kcal/mol).

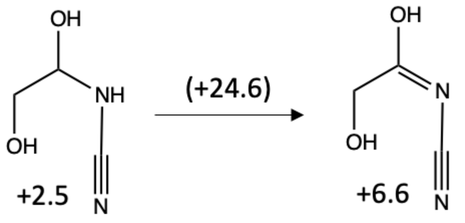

As discussed in Section 3.3 of the main article, to avoid a zwitterionic intermediate, we use the enamine tautomer shown in Figure S3 of the oxazole **12a** and the thiazole **12b** as reactants when condensed with the C_3 (glyceraldehyde) or C_2 (glycolaldehyde) sugars. Direct transition states to forming this intermediate are less favorable while the proton hop would be more favorable. This isomer is also mentioned by Ritson *et al.* in their Scheme 5 but is not along the main pathway to oxazole formation.

Figure S3. Enamine tautomer of oxazole/thiazole**.**

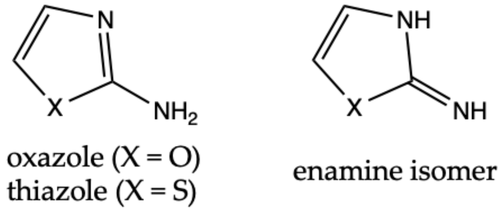

Part 5: Oxazole formation starting from glyceraldehyde

As discussed in Section 3.2, cyanamide can add to the C_3 sugar glyceraldehyde to form an oxazole. Only D-glyceraldehyde was considered and retains its stereochemistry throughout the reaction. The energetics of this pathway are shown in Figure S4. The combined reactants have a G_{rel} value of –6.6 kcal/mol.

- Step 1 is marginally exergonic (ΔG = –1.1 kcal/mol) with a barrier of 17.4 kcal/mol.
- Step 2 is rather exergonic (ΔG = –11.6 kcal/mol) and the barrier is 23.3 kcal/mol.
- Step 3 is marginally exergonic (ΔG = –1.5 kcal/mol) with a low barrier of 7.3 kcal/mol.
- Step 4 is slightly endergonic (ΔG = +4.8 kcal/mol) with a barrier of 27.4 kcal/mol.
- Step 5 is slightly exergonic (ΔG = –4.5 kcal/mol) with a barrier of 21.2 kcal/mol.

These results are not too different from those of the C_2 sugar glycolaldehyde discussed in the main article.

Figure S4. Free energies for the reaction of glyceraldehyde and cyanamide to form its corresponding oxazole (G_{rel} values in kcal/mol).

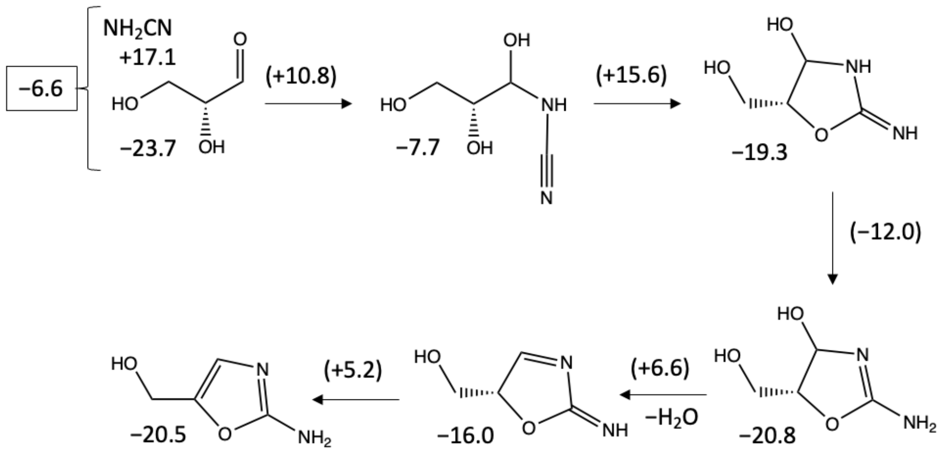

Part 6: Diastereomer Free Energies for Reaction of Glyceraldehyde and Oxazole

As discussed in Section 3.3, Figure S5 shows the G_{rel} values for all the diastereomers.

Figure S5. Free energies for the reaction of glycolaldehyde and oxazole (G_{rel} values in kcal/mol).

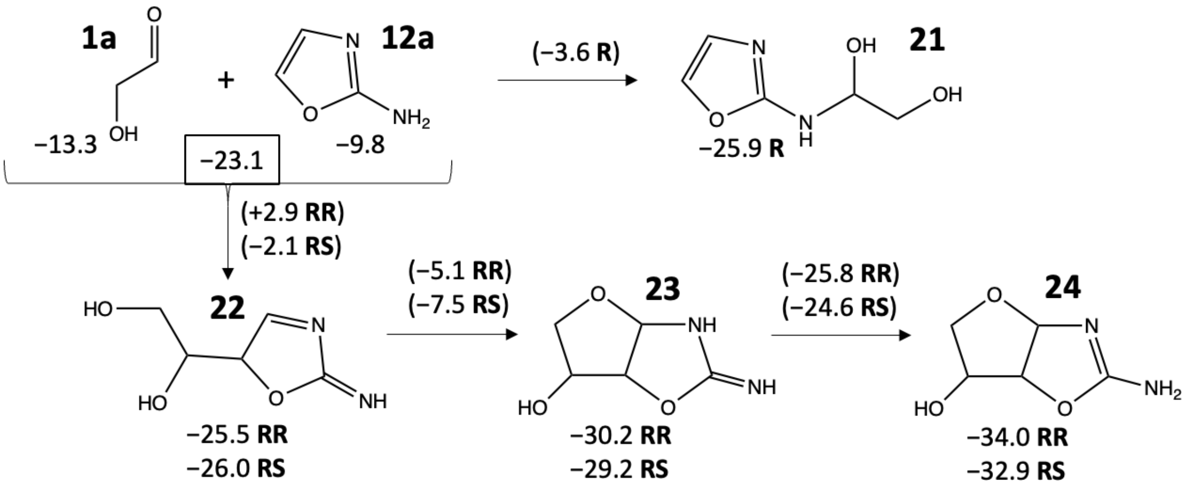

Part 7: Additional Data for the Reaction of Methylglyoxal and L-Cysteine

As discussed in Section 3.4, the G_{rel} values (in kcal/mol) for all transition state diastereomers are shown in Table S3. In two cases for **x32_33**, we were unable to find satisfactory transition states, so those numbers are unavailable.

Table S3. G_{rel} values for all transition state diastereomers.

Structure	G_{rel} (kcal/mol)
**x29+7b_30**	-64.2, -60.4
**x30_31**	-51.3, -49.7
**x31_32**	-53.2, -50.3
**x32_33**	-63.8, -63.3
**x33_34**	-64.2, -62.9
**x29+7b_35**	-65.1, -67.1
**x35_36**	-44.8, -40.9
**x36_34**	-56.9, -56.8
**x29+7b_37**	-59.5, -60.2
**x37_38**	-57.7, -57.1, -56.4, -55.6
**x38_30**	-68.8, -68.7

In Supplementary Note 1 of the experimental paper [Opfermann *et al*, *Nat. Chem. Biol.* **2025**, *21*, 1397-1407, also cited in the main article], the authors provide an approximate kinetic analysis. They cite prior experimental work estimating the first step of the reaction to form HTA has an equilibrium constant between 10^3 and 1.1×10^4. Our calculated ΔG is –11.2 kcal/mol for "1 M" concentration. Using their less concentrated reactant of 10 μM as limiting, our concentration-corrected ΔG is reduced by –(8.314)(298)/(4184 ln 0.00001) = 6.8 kcal/mol, where R = 8.314 J K^{-1} mol^{-1}, T = 298 K, and 4814 is the conversion factor from kcal/mol to J. Thus, we have an effective ΔG of –4.2 kcal/mol which corresponds to an equilibrium constant of ~1.6×10^3.

The highest energy transition state in the proposed pathway (**x30_31**) has a G_{rel} value of –51.3 kcal/mol. Since the reactants (**29** + **7b**) have a combined G_{rel} value of –67.2 kcal/mol, one estimate of the overall activation energy of the entire pathway is the difference between these two values of 15.9 kcal/mol. Not knowing the pre-exponential factor, but using a gross estimate from transition state theory of $k_B T/h = (1.38 \times 10^{-23})(298)/(6.626 \times 10^{-34}) = 6.21 \times 10^{12}\ s^{-1}$, and the Arrhenius equation: $k = (6.21 \times 10^{12}) \exp[(-51.3)(4184)/(8.314)(298)]$ yields a rate constant of $1.36 \times 10^{-4}\ s^{-1}$. We consider this a gross guesstimate, given the uncertainty of the multiple assumptions we used, and do <u>NOT</u> claim that our calculated energies match the cited experimental rate constant.

Part 8: XYZ coordinates for transition states

x1a_2a

C	-2.064613772	0.488682872	-0.055318233
C	-0.565173178	0.202797856	-0.155064374
O	-0.202712982	-0.734642418	0.741603981
O	-2.778631121	-0.710369357	-0.252983694
N	0.225715576	1.506950859	0.141823328
O	1.844572936	-1.506803399	-0.240007939
O	2.633694313	0.775061282	-0.179106433
H	-2.391435348	1.200249277	-0.821191748
H	-2.267031572	0.925659014	0.938076601
H	-0.285483851	-0.043727921	-1.192043642
H	-2.294027079	-1.370993992	0.262674988
H	1.345646382	1.266090223	0.031112028
H	-0.035100434	2.279537030	-0.470755074
H	0.033308460	1.768444379	1.108264029
H	2.324942829	-2.199961533	0.217979722
H	0.784086008	-1.220596388	0.334206338
H	3.218238163	0.914809568	0.571600068
H	2.405942761	-0.327614025	-0.225524036

x2a_3a

C	-2.145975188	0.269357173	-0.514930468
C	-0.692409431	0.640577079	-0.671806949
O	-2.391392947	-0.150339779	0.805957917
N	-0.071581631	1.307318365	0.232765511
O	2.436688302	1.189658365	0.062167187
O	2.144623863	-1.213069350	0.059285041
O	-0.249982921	-1.553714027	-0.008423385
H	-2.400965672	-0.490874731	-1.261660954
H	-2.760096206	1.157393231	-0.709566491
H	-0.174760878	0.474804275	-1.610997656
H	-1.758879224	-0.892511450	0.910447788
H	-0.561926445	1.404997744	1.118186359
H	1.043977879	1.418756118	0.160983684
H	2.888761074	1.481856644	-0.733842177
H	2.557932180	-1.625004234	0.822342749
H	2.392934637	0.061409398	0.059197815
H	-0.399756017	-2.282715222	-0.618451273
H	0.887681694	-1.432315041	0.066612061

x3a_4a

C	-2.530305606	-0.029948756	0.350220832
C	-1.210380646	0.645620202	0.075867709
H	-3.291558475	0.761622087	0.394929716
N	-0.406180251	1.124599775	0.942915105

O	1.924141823	1.692747583	0.283633667
O	2.907767736	-0.350281038	-0.534792365
H	-2.485377889	-0.492214567	1.350951629
O	-2.898742789	-0.915874008	-0.662080519
H	-0.936595210	0.736859727	-0.974057189
H	-0.679532351	0.978896836	1.913736538
H	1.973403949	2.377512897	-0.390788993
H	0.830952136	1.495747490	0.604563683
H	2.426588391	0.731943833	-0.095453232
H	3.512468212	-0.765207042	0.088524675
C	-0.303347178	-2.052808438	-0.724742870
N	0.828811188	-1.776543617	-0.742175903
H	2.092600965	-1.017206207	-0.673382579
H	-2.149419174	-1.557241584	-0.755070065

x4a_5a

C	2.471826955	-0.872989417	-0.035967840
C	1.410589774	0.200695264	0.261086370
C	0.037309644	-0.382795602	0.238210167
N	1.595240436	1.308405326	-0.679152170
N	-0.612350480	-1.347174785	0.027990016
O	3.758996121	-0.297231858	0.049162330
O	-3.077091578	-1.384988777	-0.136051277
O	-3.158491443	1.023223371	-0.290458945
O	-0.918460535	1.278699263	0.651136832
H	2.275537722	-1.303155630	-1.030838693
H	2.413086747	-1.682903968	0.693483655
H	1.595212417	0.590567814	1.265064200
H	0.958444913	2.052985197	-0.409282633
H	1.316010697	1.011798600	-1.611481176
H	3.680313389	0.562003988	-0.389954615
H	-3.339753742	-1.797432085	-0.966136354
H	-1.901331025	-1.457385095	-0.034468989
H	-3.802154734	1.483420623	0.255630264
H	-3.232600815	-0.280098475	-0.213317620
H	-2.148805976	1.212369349	0.123863508
H	-1.019759093	1.205109490	1.607186442

x5a_6a

O	3.384235914	0.818220602	0.533312497
C	1.954142836	0.908157340	0.544673684
C	1.458433930	-0.508034489	0.256835201
C	-0.042150504	-0.638212952	0.378351030
O	-0.623913373	0.055080063	1.308074198
N	-0.602772389	-1.755070227	-0.092447647
N	1.882469726	-1.056108074	-1.031153570
O	-3.009304973	-0.468641704	0.760346294
O	-2.705155841	1.342323396	-0.840922184

O	-0.400181546	0.729516265	-1.283298702
H	3.736463281	1.711446952	0.546645765
H	1.570587319	1.571066991	-0.236321901
H	1.583694201	1.246212892	1.519086646
H	1.863426975	-1.161536353	1.041986554
H	-0.085932877	-2.205342006	-0.836584732
H	-1.615374646	-1.816992447	-0.055516748
H	2.890813606	-1.163741140	-1.016281522
H	1.669842241	-0.354928624	-1.736927450
H	-3.800612008	-0.456458349	1.303762918
H	-1.635888629	-0.130471907	1.285503242
H	-3.018358835	0.360977958	0.056575109
H	-2.747453094	2.225178857	-0.463504046
H	-1.577044869	1.112395180	-1.066557578
H	-0.507755498	0.192397495	-2.076061497

x6a_7a

O	-3.539724013	0.713821616	-0.172665917
C	-2.151568287	0.838241039	-0.489875029
C	-1.399928851	-0.118519961	0.422281364
C	0.135404925	-0.034765135	0.245120275
N	-1.847472367	-1.520866268	0.334950331
N	0.562051778	-0.593040792	-1.166586404
O	0.585422355	1.187294475	0.371222607
O	0.725551371	-0.984749890	1.139663106
O	3.008948746	1.347153471	0.336749710
O	2.989290163	-0.858609249	-0.633586559
H	-4.014964946	1.417489177	-0.622177701
H	-1.770923040	1.849966347	-0.322858135
H	-1.974129318	0.569001874	-1.545551493
H	-1.559087069	0.210592667	1.451724058
H	-2.708385859	-1.630220619	0.859745100
H	-2.095137262	-1.749627252	-0.624954836
H	0.326438325	0.091077821	-1.880045329
H	0.111461472	-1.479197924	-1.385407083
H	1.887825760	1.325315752	0.417307735
H	0.073756734	-1.703117252	1.198273337
H	3.138128824	0.189695491	-0.216200792
H	3.251435358	2.070730809	-0.248118252
H	2.867845366	-1.383227128	0.170688426
H	1.860914985	-0.764058910	-1.003126463

x1b_2b

S	2.833500526	-0.308426445	-0.422649844
C	1.545993996	0.827071338	0.274447405
C	0.093671704	0.418413566	0.012644726
O	-0.742007769	1.256052077	0.643110834
N	-0.156757815	-1.024373263	0.535519348

O	-2.558354447	-1.448655476	-0.180070398
O	-2.757969748	0.916341369	-0.621363818
H	3.184314384	-0.898751980	0.736303453
H	1.694115651	1.802664962	-0.186440968
H	1.699832412	0.944600278	1.348084631
H	-0.085671973	0.319007981	-1.070647492
H	-1.232122764	-1.306212722	0.265049094
H	0.510001881	-1.692378039	0.146942460
H	-0.066279790	-1.001554162	1.550475094
H	-3.172399204	-1.730385147	0.503881217
H	-2.787896320	-0.366381546	-0.421275617
H	-3.541130895	1.387041325	-0.327238752
H	-1.752736459	1.202350393	0.081607237

x2b_3b

S	2.618701072	-0.420508092	0.318502788
C	1.538108114	1.015569181	-0.082817490
C	0.085809521	0.732118892	0.154922857
N	-0.848393533	1.507121213	-0.310290560
O	-3.131342737	0.545650336	0.182959044
O	-1.923922260	-1.526319931	0.433288461
O	0.061288306	-1.113504412	-0.929698920
H	3.550500076	0.307992730	0.967928504
H	1.692413268	1.327344859	-1.117897145
H	1.812021811	1.851235168	0.567421617
H	-0.191522571	0.093670208	0.986587909
H	-0.618148806	2.140846592	-1.066389322
H	-3.511054737	0.779560051	1.034925529
H	-1.917398105	1.226348489	-0.086698632
H	-2.666448071	-0.500357565	0.299709648
H	-2.394287978	-2.361389773	0.389767346
H	-0.822493376	-1.421712862	-0.384064573
H	-0.241251196	-0.887439509	-1.816024368

x3b_4b

S	3.052624752	-0.098841020	0.902668112
C	1.915329996	0.614686287	-0.358657982
C	0.522585447	0.736019423	0.161957402
N	-0.177651273	1.799882654	0.218902899
C	-0.883143389	-0.795493193	-2.159166799
N	-1.497403367	-1.308742226	-1.302412395
H	-2.166520269	-1.390524165	0.054072633
O	-2.610298874	-1.052138725	0.947822067
O	-2.649558207	1.143177690	-0.052682683
H	2.970121478	0.928793799	1.772367965
H	1.893908681	-0.109530534	-1.177986608
H	2.298478508	1.563111992	-0.739701029
H	0.062667060	-0.189310308	0.500208829

H	0.300070645	2.627267563	-0.139683425
H	-3.429292027	-1.536736918	1.092111785
H	-2.737869632	0.176343924	0.524913470
H	-1.705588868	1.544871816	0.173118452
H	-2.526257010	0.804854959	-0.961120185

x4b_5b

S	-0.983989207	-2.019999246	-0.309401295
C	-1.797688432	-0.495934496	0.378987876
C	-1.491284800	0.767452753	-0.457469263
C	-0.017946762	0.919473407	-0.649307595
N	-2.043609654	1.981129401	0.134023552
N	0.861643483	0.846989293	-1.437330223
O	0.436422849	1.141368697	1.200428956
O	1.986179634	-0.798309494	1.260807123
O	3.092708122	0.082927772	-0.697510860
H	-1.647655857	-2.894188202	0.471957822
H	-1.447486580	-0.329289522	1.395555665
H	-2.877071213	-0.653730203	0.373457055
H	-1.903940185	0.642769111	-1.463584324
H	-1.625826842	2.096640257	1.053201924
H	-3.049509677	1.895265944	0.237364133
H	1.317651613	0.025284001	1.354378187
H	0.960999671	1.948865085	1.168198068
H	2.667636914	-0.397530804	0.245562422
H	1.422753871	-1.535170417	0.984557716
H	3.716638382	0.778827728	-0.465599083
H	2.136792003	0.534716355	-1.135499076

x5b_6b

S	2.633799722	-0.700781543	0.115500358
C	1.690856610	0.824302044	0.561165927
C	0.157015901	0.674113671	0.662967131
C	-0.467103504	0.782773017	-0.739740995
O	-0.318601682	-0.132902870	-1.612300626
N	-0.660054673	2.045092114	-1.218488866
N	-0.350708714	1.637061107	1.633254772
O	-0.241001856	-2.359454120	-0.712460214
O	-2.057005822	-1.906466566	0.863213798
O	-2.361891282	0.392413203	0.151392481
H	2.824303994	-1.135195031	1.376847247
H	1.958076018	1.569605283	-0.189574840
H	2.060177122	1.169469247	1.525949899
H	-0.089331566	-0.320504905	1.028631983
H	-1.083893492	2.688298293	-0.568320920
H	-1.047569306	2.075869069	-2.150941181
H	-1.367523999	1.605002065	1.593759227
H	-0.038484740	2.580157635	1.422162582

H	0.625536507	-2.447669972	-0.296061735
H	-0.244044639	-1.234201330	-1.191990217
H	-0.999533373	-2.290607760	0.045036664
H	-1.990737673	-1.977563851	1.818282834
H	-2.979581757	0.396242283	-0.586700583
H	-2.274557000	-0.826594187	0.568167405

x6b_7b

S	3.672253874	0.527250442	0.125039079
C	1.857576614	0.696195781	0.451506173
C	1.076552093	-0.281970493	-0.421196936
C	-0.457833497	-0.094849064	-0.243199798
N	1.432297529	-1.702851436	-0.250479170
N	-0.904618159	-0.556225422	1.191550972
O	-0.831183627	1.141588528	-0.436429544
O	-1.106608653	-1.050121155	-1.094001118
O	-3.246409344	1.448304392	-0.387598295
O	-3.346015752	-0.715482805	0.671575222
H	4.020955738	1.762602126	0.532112483
H	1.527055126	1.705608444	0.215997201
H	1.675930256	0.499653980	1.512407273
H	1.240939136	-0.022139801	-1.469096244
H	2.193763922	-1.966309816	-0.865134597
H	1.745716954	-1.897371759	0.695941524
H	-0.628736003	0.151598329	1.866478420
H	-0.491917695	-1.449819798	1.451479377
H	-2.142052869	1.362336921	-0.478507716
H	-0.522893978	-1.825822817	-1.089882974
H	-3.439121442	0.319671861	0.217303437
H	-3.441430970	2.214639628	0.159307691
H	-3.250103705	-1.274653982	-0.113178698
H	-2.227828133	-0.668370398	1.038003447

x1a_8a

C	1.584847236	1.580409553	-0.151562722
C	0.243602193	1.012994553	0.292071611
C	0.954988664	-1.617869408	0.181861024
N	1.504437353	-2.279239843	0.961934155
N	0.268647256	-0.777334277	-0.602207045
O	-0.785729299	1.647781707	-0.086525266
O	2.675681969	0.684341888	-0.179376654
O	-2.767415082	0.554680091	0.769475633
O	-2.211174746	-1.255217696	-0.693876625
H	1.771416081	2.442180700	0.510178437
H	1.461047413	1.969476710	-1.164150711
H	0.240183669	0.517455282	1.275954429
H	-1.111083792	-1.096697600	-0.714208708
H	0.768153982	-0.619496538	-1.475099433

H	2.754378626	0.236428435	0.670852714
H	-3.589349473	1.039219237	0.650926912
H	-1.910082432	1.163578742	0.442488312
H	-2.549320776	-1.156424712	-1.589784709
H	-2.618088934	-0.397708996	0.023675088

x8a_9a

C	-1.190002621	-1.302007469	0.317940000
C	-2.160910182	-0.120607674	0.268671760
N	-1.350958417	1.012036128	0.730069346
C	-0.067531844	1.105353241	0.320200061
N	0.938300943	1.712771177	0.205471910
O	-0.048551141	-0.815468839	-0.318435726
O	-2.679279854	0.081826416	-1.027190182
O	2.232442736	-1.551960489	0.097628810
O	3.103551066	0.659221472	-0.345022612
H	-1.641911420	-2.152455599	-0.211273509
H	-1.013658284	-1.594197427	1.367186239
H	-3.019569748	-0.220980237	0.933227939
H	-1.822893988	1.905338559	0.777907020
H	1.176830773	-1.323506928	-0.062506523
H	-1.928526328	-0.018359767	-1.628795061
H	2.787152736	-0.403323571	-0.136262542
H	2.348562283	-1.810917244	1.016573975
H	2.149838835	1.244964743	-0.104990544
H	3.284646150	0.747972740	-1.287301606

x9a_10a

C	2.304789700	-0.337266775	0.061570926
C	1.366264186	0.816870095	0.429377520
O	1.391253163	-1.433141566	-0.264834054
O	1.058269599	1.574725693	-0.701933584
N	0.200652174	0.050696101	0.891256900
C	0.184535185	-1.090608701	0.181175481
N	-0.874568585	-1.807345869	-0.073046156
H	2.935604128	-0.657632930	0.892413806
H	2.891722187	-0.118668075	-0.827017726
H	1.755236947	1.430146135	1.250095603
H	0.103311532	1.841862619	-0.619040332
H	-0.665445217	0.634152389	0.898818938
H	-0.720676394	-2.616290501	-0.658205161
O	-1.538743441	1.749376728	-0.028749279
O	-2.855402258	-0.268684803	-0.235916384
H	-2.309515026	0.763104097	-0.205832047
H	-2.027640323	2.540626734	0.209287695
H	-3.487501795	-0.282158034	0.488527826
H	-1.861538158	-1.221357486	-0.102880506

x10a_11a

C	-2.140490075	-0.173417937	0.201758681
C	-1.211489798	0.499727590	-0.799122234
O	-1.275299749	-1.123700600	0.837413170
N	-0.143064704	-0.318134829	-0.978618252
H	-1.663196804	0.940814042	-1.685420386
C	-0.100780975	-1.150511191	0.101776572
N	0.919967169	-1.845481569	0.465203746
H	0.703887159	-2.422745330	1.271926412
H	-2.542899244	0.508878354	0.949444768
H	-2.954375783	-0.695833989	-0.311961780
O	-0.726463629	1.914128719	0.002161952
O	1.511624287	1.469184058	0.865259963
O	2.524807988	-0.055680295	-0.776564299
H	0.471477418	1.694292969	0.468199648
H	2.041477950	0.824828312	0.138241814
H	2.332106226	-0.908506069	-0.300006365
H	1.406481286	0.935628044	1.662672275
H	1.711038223	-0.047340363	-1.338797446
H	-0.666066986	2.612958934	-0.659479212

x11a_12a

C	-2.084297550	-0.097253600	-0.084448717
C	-1.252711573	-0.554188590	-1.121247705
O	-1.442558525	-0.564874113	1.100549738
N	-0.100473113	-1.019533695	-0.668836798
H	-1.440010273	-0.443467128	-2.182865364
C	-0.192129031	-0.983266240	0.715788777
N	0.790182833	-1.172129257	1.526080108
H	0.457880317	-1.097971211	2.486053096
H	-1.465756570	1.292569064	-0.052734408
H	-3.165582264	-0.137587115	-0.059228844
O	-0.746393525	2.170132431	0.164040776
O	1.534111990	1.416276941	0.591677644
O	2.343124834	-0.072274620	-1.454887090
H	0.312797856	1.836641433	0.357855669
H	1.943592952	1.041954102	-0.237508405
H	3.050641135	-0.645252800	-1.141023299
H	1.462178558	0.600005633	1.145346769
H	1.517202463	-0.604680357	-1.325617601
H	-0.763648887	2.787794891	-0.575796814

x9a_11a

C	-2.084488191	0.533409521	0.048564397
C	-0.903422260	0.990225242	-0.797125825
O	-1.638584374	-0.734030920	0.546738971
N	-0.152134400	-0.106510564	-1.074254647
H	-1.109083860	1.678943192	-1.614006610

C	-0.478190809	-1.050611922	-0.143701706
N	0.220984942	-2.094104217	0.136593478
H	-0.245443124	-2.670491339	0.830300765
H	-2.303908797	1.196502092	0.884624776
H	-2.977499033	0.397711636	-0.570668884
O	-0.054838264	2.027309808	0.238765128
O	1.808764840	0.722437482	1.122160509
O	2.424996598	-0.765770017	-0.735725539
H	0.948312814	1.347891593	0.726137140
H	2.166344952	0.062909051	0.309391195
H	1.692630538	-0.410404014	-1.301302619
H	1.452488278	0.134343475	1.799529243
H	1.949404515	-1.579631850	-0.422422279
H	0.304094243	2.741670127	-0.300641463

x10a_12a

C	0.448091967	0.644584814	-1.038807044
C	0.228528233	-0.799206648	-0.970645155
N	-0.959182118	-1.088806537	-0.399196441
O	-0.788211503	1.165243862	-0.453845899
C	-1.467012729	0.096876255	-0.082464558
N	-2.617830561	0.277897364	0.571581537
O	1.708625186	-1.407226832	0.457628256
H	0.590731034	1.071818597	-2.030404629
H	1.356133466	0.949010013	-0.265804768
H	0.753053059	-1.531322134	-1.559981191
H	-2.917058138	1.204998287	0.819996477
H	-3.101756092	-0.526852755	0.930235953
H	1.154478193	-1.715395540	1.182763806
O	2.258044755	0.913865021	0.834713654
H	2.073815240	-0.398451787	0.732982402
H	3.142962477	1.164714193	0.550685190

x1b_8b

S	-2.370895938	-2.123194731	0.736075768
C	-1.521359443	-0.898593915	-0.352298403
C	-0.098559772	-0.743069490	0.111354427
O	0.754632505	-1.604564112	-0.231956799
O	2.872784992	-1.078550550	0.837579816
O	2.743724844	1.185571894	0.072193968
N	0.412032469	1.005768555	-0.909028724
C	-0.434193805	2.000675259	-0.640126265
N	-1.170724842	2.828665549	-0.288099684
H	-1.566550158	-3.158295655	0.420588478
H	-2.082424186	0.029000572	-0.241948369
H	-1.547562851	-1.235765370	-1.388125726
H	0.022579977	-0.198616674	1.058262132
H	2.852748836	-1.239598008	1.785899309

H	1.930573538	-1.418262138	0.380567149
H	2.896517055	0.077658695	0.543713403
H	2.788794813	1.908205177	0.706276260
H	1.750604943	1.180632463	-0.387941580
H	0.397659397	0.744282568	-1.892519303

x8b_9b

S	0.099920604	-1.320374048	-0.908654350
C	-1.613939195	-1.203715102	-0.282829877
C	-1.874554163	0.090573622	0.486592051
N	-1.363071773	1.288868707	-0.265552402
C	-0.053313180	1.471591810	-0.235523926
N	1.005474748	1.943073397	-0.057784783
O	-3.252511053	0.247248134	0.666092240
O	2.418273542	-1.435475863	1.036484409
O	3.136720886	0.710531198	-0.036028350
H	1.569327878	-1.582466478	0.498682581
H	-1.871635803	-2.033413283	0.379622933
H	-2.314958037	-1.226814938	-1.123319237
H	-1.320256469	0.090536715	1.433604086
H	-1.746588235	1.321101554	-1.208006586
H	-3.406203336	1.010382967	1.233780634
H	2.906189154	-0.189001821	0.445678802
H	2.133132483	-1.406738515	1.955635641
H	2.142292052	1.356600858	-0.092478194
H	3.831221700	1.169165628	0.448779094

x9b_10b

C	2.288401026	0.257138060	-0.018095578
C	1.051211882	1.083206422	0.356462838
S	1.635292088	-1.315867222	-0.726974463
C	0.045082808	-1.003494812	-0.017888688
N	0.020068255	0.108948596	0.727522489
N	-1.008968794	-1.753846297	-0.215164611
O	0.634369682	1.853190065	-0.729489954
O	-1.928969675	1.714473564	0.121974590
O	-3.116678236	-0.378670665	-0.120713140
H	2.900024181	0.027127240	0.853502133
H	2.869410601	0.776840728	-0.776879621
H	1.262601144	1.702994877	1.237779162
H	-0.902119113	0.596999256	0.839720510
H	-0.898259554	-2.564615255	-0.806175330
H	-0.334127636	2.021905734	-0.592188178
H	-2.431944195	2.428966379	0.520044734
H	-2.649711328	0.682053712	-0.062343138
H	-3.670508170	-0.488989723	0.657723301
H	-2.030408182	-1.239238997	-0.123707589

x10b_11b

C	-2.046487584	0.722229136	-0.257720330
C	-0.692099786	1.044314311	-0.895748259
S	-1.739427169	-0.804218200	0.694684920
N	0.104193971	-0.041570236	-1.086580943
H	-0.764418558	1.711539180	-1.754954535
C	-0.145898509	-1.044007448	-0.214503693
N	0.670139259	-2.014076673	0.029643799
H	0.284657924	-2.710286053	0.658672054
H	-2.390772617	1.526887601	0.388638453
H	-2.780777285	0.532791128	-1.043131604
O	0.017191707	2.069773853	0.210975347
O	1.820324652	0.812118087	1.252677946
O	2.777129118	-0.458477700	-0.664673375
H	0.978009127	1.425108813	0.784010999
H	2.321448210	0.291622720	0.454795937
H	2.350454652	-1.323287749	-0.428980534
H	1.394328151	0.117144171	1.772870445
H	2.075817519	-0.140429027	-1.275921104
H	0.390774124	2.810702428	-0.279959386

x11b_12b

C	-1.958806197	0.345717698	-0.515009279
C	-0.986417356	-0.023107999	-1.460884899
S	-1.622766604	-0.620880571	0.963085665
N	0.073675378	-0.704648245	-1.069907904
H	-1.029603557	0.324629610	-2.489865323
C	-0.010952549	-1.099116270	0.251087769
N	0.986831581	-1.612076168	0.888394305
H	0.738063422	-1.875488671	1.838090502
H	-1.143918533	1.637703083	-0.140108940
H	-2.990033671	0.539532715	-0.784344446
O	-0.405531251	2.396578551	0.236859301
O	1.722080831	1.350994363	0.812676983
O	2.781736323	-0.250066727	-0.976974493
H	0.573006515	1.923346999	0.477368042
H	2.208755099	0.893792817	0.054014674
H	2.799100828	-0.975723619	-0.331005082
H	1.520692938	0.591947929	1.383128873
H	1.914599756	-0.441768679	-1.399007265
H	-0.287738418	3.082884180	-0.430145058

x13+12a_19

O	-2.266045990	-3.311125012	0.238456700
C	-2.424066932	-1.904734261	0.251696049
C	-1.070476761	-1.216463737	0.052839956
C	-1.249036739	0.294121178	0.033251388
N	0.575004834	1.005323587	0.362546503

C	1.689282048	0.236487155	0.174219852
O	2.657024935	0.228906065	1.130171614
C	3.626484759	-0.630239597	0.646093033
C	3.204059958	-1.091466230	-0.547789643
N	1.960815806	-0.536982308	-0.843667234
H	-1.739958658	-3.505439261	-0.547176392
H	-2.842672052	-1.617428551	1.216074560
H	-3.111794541	-1.568740882	-0.539601065
O	-0.574834818	-1.669066270	-1.196720658
H	-0.415133120	-1.514397253	0.881690424
O	-1.961309319	0.871771213	0.915714447
H	0.495002496	1.267784160	1.342970793
H	4.494087491	-0.777535267	1.263791375
H	3.688155176	-1.784053321	-1.216590959
H	0.359610556	-1.391884432	-1.277577637
H	-1.258026501	0.682241946	-0.995334968
H	-2.164074905	2.096053757	0.530074728
O	-2.154558232	3.136666748	0.087980634
O	0.034069903	3.091338165	-0.897101946
H	0.399651788	2.138346447	-0.374711137
H	-1.099212504	3.203744577	-0.483374774
H	-2.915171151	3.263310052	-0.486402383
H	0.073222776	2.959740297	-1.849783959

x13+12a_14

C	-0.105441096	-1.261772874	-1.030109407
C	-1.310790396	-0.631322095	-1.564995996
O	-0.513863472	-1.650417302	0.289321363
N	-2.119787220	-0.345499398	-0.601500432
H	-1.450676770	-0.261989061	-2.572163223
C	-1.598022344	-0.911163241	0.602375795
N	-2.163159654	-0.716942218	1.713897505
H	-1.648960736	-1.156350761	2.472989360
C	0.954981625	-0.002882090	-0.866596748
C	2.363098905	-0.650418598	-0.639551995
C	3.268936350	0.355502856	0.083590319
O	0.522834429	0.794022166	0.127253143
H	2.778051608	-0.908270873	-1.620652151
O	2.312935036	-1.859012752	0.098550943
H	4.285769751	-0.042449452	0.125487434
O	2.820531003	0.513810058	1.422301782
H	3.287017357	1.310962687	-0.460359131
H	2.216760301	-1.590315743	1.023165309
H	1.891076739	0.805230010	1.312620776
H	0.984106282	0.493238358	-1.859969727
H	0.343882672	-2.101059703	-1.551853415
O	-3.299094383	1.636301145	0.202404709
O	-1.082498066	2.624342847	-0.400971493

H	-0.362137051	1.896045741	-0.176865026
H	-2.447289303	2.207086431	0.052285904
H	-2.884445608	0.615583991	-0.364336661
H	-0.708273578	3.486841542	-0.206390266
H	-3.273276830	1.329324179	1.124165105

x14_15

C	1.359000548	-0.333338974	-0.451026831
C	1.603644792	1.056545199	0.157678594
C	0.293993729	1.461360509	0.834360750
C	-0.396283085	0.271198099	1.465556730
N	-1.667353442	0.229578595	1.081072485
O	0.583625520	-1.051409131	0.494416356
O	-0.668257095	1.856350909	-0.171330779
C	-1.773755555	1.051300555	-0.022009380
C	2.671193325	-1.079684656	-0.696135986
O	3.315816537	-1.366847172	0.535893031
N	-2.759141298	1.030183190	-0.838696628
H	0.818451002	-0.214958294	-1.404195603
H	0.433490838	2.291130922	1.531542890
H	-0.103650339	-0.116594319	2.431758796
H	2.464070044	-1.999649229	-1.261840213
H	3.345002425	-0.453006356	-1.285351913
H	2.615339016	-1.711883537	1.105598148
H	-2.650840213	1.704867377	-1.589052890
O	2.070868713	2.011546571	-0.770457289
H	-2.872214941	-1.024600426	0.729559443
H	2.374490035	0.958462538	0.926308717
H	1.337828351	2.205240812	-1.366984590
O	-1.104358688	-2.343842936	-0.741157313
O	-3.258985722	-1.479087020	-0.079683803
H	-0.353009780	-1.810022369	-0.161989238
H	-2.268174730	-1.960743782	-0.457169890
H	-0.967277729	-3.290339920	-0.642709467
H	-3.378948923	-0.645421558	-0.629808134

x15_16

C	1.644860417	-0.361778648	-0.626227527
C	1.932988431	0.880141628	0.236184080
C	0.678756023	0.962052068	1.118178343
C	0.121145597	-0.483423078	1.129449265
N	-1.213346719	-0.330119244	0.614879933
O	0.979318756	-1.252605933	0.279522493
O	-0.339642218	1.731781042	0.428549119
C	-1.396176975	0.906267979	0.177340621
C	2.887955166	-1.066207536	-1.144670333
O	3.726570443	-1.493377599	-0.081898184
N	-2.447592163	1.374380832	-0.435429667

H	0.991047646	-0.089306181	-1.468746484
O	2.226698082	2.049649506	-0.489367752
H	2.801224767	0.656720396	0.859677711
H	0.836247251	1.416229742	2.094884280
H	0.123420117	-0.984728564	2.097320951
H	3.464706120	-0.361981700	-1.748694267
H	2.588694620	-1.908806402	-1.781976750
H	3.174434045	-2.039752444	0.489575378
H	-2.418727855	2.365296902	-0.627869620
H	-3.380288297	0.618538874	-0.748885724
H	1.393326336	2.381188866	-0.844992625
O	-3.076104116	-1.940539832	0.311596554
O	-4.225550448	-0.271192287	-0.977142952
H	-2.090163167	-1.200415924	0.510253028
H	-3.738717210	-1.184776963	-0.350625005
H	-3.540761157	-2.111971315	1.136189277
H	-4.217203687	-0.504119424	-1.910432698

x14_17

C	0.457131926	1.019562034	1.175038860
C	1.914936575	0.574080520	0.923860807
C	2.143820958	0.251588061	-0.567026384
C	1.023400878	-0.612043625	-1.175933285
C	-0.328635649	0.059976847	-1.284623779
O	-0.107401914	1.572456326	-0.006063854
N	-1.258663699	-0.763207732	-0.841926498
C	-0.649810824	-1.819266645	-0.166054880
O	0.722261812	-1.763333293	-0.350922068
N	-1.272428985	-2.678609485	0.519102570
H	0.412682125	1.750346629	1.990070864
H	-0.124941842	0.145403100	1.505779923
O	2.287200310	-0.501634472	1.773896293
H	2.602307552	1.390565671	1.156778450
O	2.265931572	1.425380429	-1.353718998
H	1.333683889	-0.957274877	-2.168040728
H	-2.567699309	-0.413569547	-0.461749059
H	-0.634155843	-3.368399429	0.908930512
H	-0.544822747	0.791411209	-2.049043463
H	1.796859054	-1.276280316	1.473268038
H	3.087344051	-0.292445666	-0.642662677
H	1.502071338	1.969774966	-1.103929397
O	-3.472898215	0.012338667	0.016130857
O	-2.410329733	2.087576786	0.595785567
H	-3.055162116	1.042462548	0.343355634
H	-1.355412092	1.927719513	0.290862584
H	-3.659760158	-0.551284122	0.776760270
H	-2.764593735	2.878777770	0.180829268

x17_18

C	1.085762628	0.399104931	1.581662507
C	2.221846445	-0.455967034	0.962991576
C	2.388557227	-0.177060338	-0.544658183
C	1.045216024	0.120188091	-1.205800048
C	0.240036828	1.225416476	-0.473290697
O	0.773608399	1.544420947	0.795959990
N	-1.088314672	0.649050339	-0.419433572
C	-1.035475657	-0.642128940	-0.700680679
O	0.199642837	-1.059574908	-1.107698866
N	-2.006491518	-1.509816559	-0.612556917
H	0.198332821	-0.233549281	1.716877610
H	1.370852370	0.776754837	2.562339045
O	2.012838115	-1.841862294	1.212875139
H	3.177956283	-0.210335826	1.425487770
O	3.248447226	0.946368429	-0.706807557
H	1.167246495	0.355734284	-2.266673226
H	-1.780011884	-2.435133346	-0.947578004
H	0.244703015	2.170230429	-1.014058901
H	1.230370047	-2.106345231	0.714802733
H	2.821511576	-1.082783592	-0.985630900
H	3.570648384	0.956243689	-1.613060483
H	-3.094943235	-1.184325106	-0.132656673
O	-3.243410158	1.541638581	0.428674924
O	-4.131023862	-0.689612930	0.378223366
H	-2.121462520	1.182191756	0.003066061
H	-3.791337810	0.469441340	0.444517769
H	-3.684028415	2.108466407	-0.211251637
H	-4.246896675	-1.022978660	1.273152884

x13+12b_20

O	-2.493526588	-3.330706466	0.410792279
C	-2.609648215	-1.917821248	0.376259065
C	-1.254785405	-1.279347897	0.080349413
C	-1.380254630	0.245694664	0.020080897
N	0.233205275	0.930050850	0.325599890
C	1.424262190	0.256496559	0.049825895
S	2.812329781	0.316149091	1.133752123
C	3.688728130	-0.673702377	-0.000072700
C	2.899383766	-0.961144314	-1.065911169
N	1.625507541	-0.437211951	-1.034649781
H	-2.022185460	-3.573300161	-0.395329643
H	-2.970487334	-1.583215571	1.348005394
H	-3.328874915	-1.591089948	-0.389871919
O	-0.844586923	-1.801220554	-1.179149645
H	-0.558067663	-1.576894072	0.876917349
O	-2.139503456	0.829227360	0.895808585
H	0.140005218	1.148563535	1.315967632

H	4.705136569	-0.977704601	0.193140810
H	3.195615022	-1.567705569	-1.910168364
H	0.045314901	-1.458492027	-1.370152388
H	-1.479889277	0.565900689	-1.030001117
H	-2.427044369	2.012682059	0.472461099
O	-2.492579575	3.046049164	0.003516519
O	-0.248922397	3.059496885	-0.864541867
H	0.087895927	2.027610561	-0.315415609
H	-1.355381261	3.154338721	-0.534446087
H	-3.232396554	3.077600088	-0.609128438
H	-0.186746515	2.959163845	-1.819864844

x1a+12a_21

H	0.981981620	-3.029987580	-0.587533946
C	0.364808102	-2.163618816	-0.328008923
C	1.326183093	-0.994969578	-0.187120954
N	0.141711860	0.618602962	-0.346309617
C	-1.204844223	0.530098126	-0.137204024
O	-2.056962854	1.103030110	-1.030553636
C	-3.320646162	0.834832563	-0.535192783
C	-3.171307297	0.125781244	0.601461416
N	-1.815499422	-0.070479198	0.850132238
O	-0.289908025	-2.451199296	0.883794429
H	-0.318983201	-1.985997368	-1.169676705
O	2.227053277	-0.788153898	-1.059063928
H	0.330197374	0.900256229	-1.306029655
H	-4.152325335	1.210394582	-1.103650078
H	-3.929369317	-0.267919563	1.258683173
H	-0.899353241	-1.713403837	1.076707320
H	1.537747610	-0.762810496	0.866142903
H	3.030354580	0.096868532	-0.596926651
O	3.581740775	0.950202471	-0.074177743
O	1.713472042	1.964374973	1.043227626
H	0.890245624	1.399569492	0.465652663
H	2.733728517	1.501801930	0.562535311
H	4.299222597	0.607862998	0.466628634
H	1.621723566	1.778110255	1.982989308

x1a+12a_22

C	-0.964383665	-0.488232634	1.153060593
C	0.367210701	-0.232600715	1.691025827
O	-0.723068686	-1.507891461	0.177600025
N	1.259660058	-0.717322961	0.894536259
H	0.616414776	0.435916547	2.504025961
C	0.595388701	-1.467576422	-0.121042811
N	1.230645551	-1.991445870	-1.076524620
H	0.599874020	-2.455257886	-1.725653294
C	-1.335306022	0.922369464	0.333729306

C	-2.797376426	0.815326840	-0.154036827
H	-3.103059080	1.838447777	-0.427004985
O	-0.498993129	1.048988068	-0.699801656
H	-3.470483471	0.457444731	0.632342256
O	-2.884248096	-0.060799652	-1.252063237
H	-2.047037066	0.082611647	-1.722215179
H	-1.278576590	1.711773126	1.112391795
H	-1.761972984	-0.779224335	1.831314014
O	3.236383717	0.235419230	-0.183683716
O	1.670361619	2.155787152	-0.317873056
H	0.712249371	1.731076908	-0.512446625
H	2.704981113	1.120833708	-0.360371631
H	2.378841555	-0.294527071	0.531025736
H	1.753590346	2.965518513	-0.826615886
H	3.169282426	-0.308027314	-0.983715758

x22_23

C	-1.283619247	-1.390082623	0.479734291
C	-2.266145624	-0.352803186	-0.075434997
C	-1.364512874	0.669036593	-0.772299827
C	-0.165931729	-0.043872660	-1.385023221
N	0.955620669	0.617962700	-1.029076031
O	-0.287185203	-1.558415882	-0.526508934
O	-0.739720604	1.502863518	0.221899322
C	0.638795749	1.375232102	0.063675612
H	-1.778393641	-2.343572536	0.682515103
N	1.483994739	1.878019957	0.885169190
H	-0.844312668	-1.014232142	1.413847122
H	-1.905645282	1.297021492	-1.483264523
H	-0.230362452	-0.448467053	-2.388555476
H	1.027613171	2.420301187	1.611514024
O	-3.123123133	0.202853376	0.895145021
H	2.658072201	0.186961981	-0.705518166
H	-2.904532834	-0.821572790	-0.829451476
H	-2.584003894	0.790387359	1.438912769
O	1.827826752	-1.917358146	0.625743890
O	3.240471060	-0.035958177	0.079339369
H	0.876333835	-1.777877795	0.055260353
H	2.568571070	-1.030492248	0.427777616
H	2.226684725	-2.763460640	0.400664091
H	2.945683883	0.717534158	0.660172039

x23_24

C	-1.871955263	-0.932122777	1.106703092
C	-2.575344380	-0.030640597	0.076568872
C	-1.471544679	0.141383080	-0.984983909
C	-0.547869704	-1.085722840	-0.771396675
N	0.727996136	-0.486806866	-0.466486316

O	-1.112960047	-1.834204444	0.306019057
O	-0.649229492	1.281671337	-0.633020551
C	0.603922420	0.824471640	-0.342218540
H	-2.561317391	-1.512813435	1.717924379
N	1.530542658	1.672620265	0.009811358
H	-1.231277359	-0.331417025	1.769322850
O	-3.102343295	1.173617674	0.576438000
H	-3.405659460	-0.583325407	-0.369831250
H	-1.836189102	0.291340858	-1.999359385
H	-0.474362420	-1.775754587	-1.611877479
H	1.251864845	2.642574298	-0.022242183
H	2.649821976	1.274130896	0.344612916
H	-2.358271058	1.770883212	0.719896787
O	2.961121031	-1.470862277	-0.013943175
O	3.727591260	0.706719649	0.648804315
H	1.802146297	-1.063431697	-0.264563368
H	3.451527499	-0.431284304	0.339199073
H	3.398315435	-1.761762199	-0.819816479
H	3.856271634	0.737498990	1.601629085

x1a_12b_25

H	1.513142820	-2.899532571	-0.879854249
C	0.774170432	-2.177914239	-0.523126587
C	1.551548398	-0.897368648	-0.232976305
N	0.354080490	0.476280367	-0.273083036
C	-1.005975375	0.317127967	-0.020897980
S	-2.246137591	1.143081582	-0.966301545
C	-3.454568715	0.407476644	0.050403873
C	-2.854873801	-0.370581628	0.986651465
N	-1.478969515	-0.421039270	0.943457723
O	0.176059609	-2.721957023	0.633587380
H	0.058506256	-2.005265293	-1.340053417
O	2.458725265	-0.538491611	-1.079964696
H	0.528471360	0.816541157	-1.216761097
H	-4.505470025	0.587771282	-0.110460000
H	-3.376268149	-0.947388233	1.737653742
H	-0.467844196	-2.071195894	0.959685900
H	1.786544395	-0.822002394	0.840550695
H	3.202160633	0.345745998	-0.548775676
O	3.705097110	1.199916859	0.035248988
O	1.729390293	1.947235861	1.183568493
H	0.963062592	1.265796884	0.525092818
H	2.759447334	1.648336366	0.717654773
H	4.440034592	0.859421805	0.552991664
H	1.686654954	1.685245251	2.108816407

x1a+12b_26

C	-0.729229316	-0.181993645	0.797518720

C	0.659298232	0.194887776	0.981308783
S	-0.679152998	-1.884687901	0.109693292
N	1.557405311	-0.580019308	0.463867589
H	0.956822018	1.174993420	1.336493498
C	1.101314274	-1.804923632	-0.107748537
N	1.928721374	-2.579885960	-0.658326632
H	1.490116615	-3.412871428	-1.044268998
C	-1.349929369	0.973466094	-0.239908428
C	-2.874128924	0.789108285	-0.249629502
H	-3.273648028	1.465489554	-1.015020932
O	-1.049916101	2.216296978	0.165060700
H	-3.251578654	1.121699064	0.719106119
O	-3.326315675	-0.549891172	-0.428355539
H	-1.329667593	-0.126497627	1.704540118
O	3.394923219	1.141067640	-0.155376269
O	1.300218218	2.658472847	-0.173117283
H	0.086961571	2.481530331	-0.064031824
H	2.640792978	1.818007008	-0.240436295
H	2.516951880	-0.157078340	0.252875271
H	1.466256797	3.544306141	0.161739489
H	3.828136824	1.101206963	-1.012683432
H	-3.117574580	-0.823112931	-1.327316142
H	-0.915799536	0.681364403	-1.217095698

x26_27

C	1.425740905	-1.104394776	-0.968573483
C	2.335950356	-0.065402035	-0.319611921
C	1.380051082	0.836174986	0.488323392
C	0.319134217	-0.057265257	1.143137908
N	-0.946387182	0.353585114	1.055676634
O	0.575411447	-1.534015853	0.086315269
S	0.406471125	2.008424491	-0.531092236
C	-1.155120877	1.255436535	0.048213324
N	-2.321306174	1.503986428	-0.429916757
H	0.857136413	-0.669358358	-1.799350453
H	1.945241027	1.377703143	1.247029646
H	0.602102615	-0.565886030	2.058190522
H	-2.320939378	2.207640318	-1.159996224
H	2.923664523	0.501723741	-1.042791231
H	-2.494712277	-0.532941952	1.125482610
O	3.263158598	-0.682641728	0.566138350
O	-1.630565423	-2.202861178	-0.729103026
O	-3.206847610	-0.845931261	0.490892616
H	-0.634347084	-1.928869524	-0.352830368
H	-2.492379727	-1.557344202	-0.163687131
H	-1.759539278	-3.150472525	-0.624717583
H	-3.276519379	0.012722956	-0.021280631
H	2.024685978	-1.942302890	-1.340826072

H	2.793314705	-1.401222129	1.006256007

x27_28

C	-1.882570839	-0.869278339	1.292665748
C	-2.621280303	0.104170916	0.371782437
C	-1.514864042	0.467939906	-0.627243032
C	-0.570565480	-0.782725317	-0.608751616
N	0.785531923	-0.367366475	-0.366922841
O	-1.071752731	-1.642035757	0.415269300
S	-0.504351078	1.900586822	-0.090335262
C	0.983608793	0.892500887	-0.029379748
N	2.141541547	1.405085681	0.308616327
H	-1.275378011	-0.318945013	2.024630330
H	-3.012805460	0.976371469	0.907791042
O	-3.636829607	-0.529437349	-0.398672129
H	-1.935696857	0.666142962	-1.610079341
H	-0.622281608	-1.358123466	-1.533813434
H	2.162254742	2.400340626	0.475262842
H	3.142789733	0.711078311	0.420817521
O	2.789955719	-1.824815913	-0.603004549
O	4.098134283	-0.122908893	0.469002530
H	1.726175876	-1.137851619	-0.499661407
H	3.542113980	-1.051983949	-0.071967738
H	3.045712657	-1.908391922	-1.526637226
H	4.278779970	-0.370645050	1.380840454
H	-2.549456224	-1.549611323	1.826323228
H	-4.201193671	-1.037251537	0.191946786

x29+7b_30

C	-2.993042367	-0.790793353	0.415102683
C	-2.055927932	-0.162729732	-0.605921464
C	-0.639342447	-0.719622651	-0.294003671
S	0.641437827	-0.089468464	-1.449742669
C	1.935384590	0.925371887	0.427476102
C	1.119446748	2.199310572	0.248763230
C	1.606176215	3.170656480	-0.805680478
O	-3.361077848	-0.277505662	1.437747625
O	-3.323804650	-2.061290333	0.067213735
O	1.775670947	0.223142569	1.445820452
O	0.176669025	2.424171054	0.972902224
N	-2.208847486	1.277977933	-0.561533496
H	-0.648873438	-1.810893794	-0.346655569
H	-0.340702708	-0.422200825	0.718336404
H	2.854636457	0.850863336	-0.173889849
H	2.388664003	3.798472942	-0.364896959
H	2.023169336	2.660624727	-1.675320176
H	0.782224634	3.814836784	-1.110949683
H	-3.876142027	-2.408488619	0.783455648

H	2.483044247	-1.051153535	1.454117488
H	-2.350601189	-0.521971997	-1.595265526
H	-1.982045783	1.623724432	0.366741342
H	-1.552535417	1.706747017	-1.205446016
O	2.889167013	-2.000214745	1.303978298
O	2.210656821	-2.469172145	-0.964577666
H	1.641013832	-1.565097732	-1.243601209
H	2.558550282	-2.319046829	0.099720888
H	2.953945529	-2.566636470	-1.571678307
H	2.517153164	-2.583728949	1.973352224

x30_31

C	-3.149688162	-0.306365105	0.126450386
C	-2.225206389	0.379378074	-0.871061086
C	-1.036379663	-0.497154206	-1.293290168
S	0.021620960	-1.124438026	0.073210024
C	0.883786913	0.361704337	0.641649033
C	1.673315643	1.070795029	-0.303278027
C	1.876453953	2.551248075	-0.066386324
O	-3.636530645	0.228639917	1.087887952
O	-3.419133475	-1.577011119	-0.236442782
O	0.241438918	1.112615342	1.629392685
O	2.285016151	0.545074099	-1.286833945
N	-1.795142716	1.656122883	-0.294665464
H	-0.400978028	0.051390038	-1.993208712
H	-1.413094948	-1.384878798	-1.802748546
H	2.101314829	-0.415752549	1.308441116
H	0.920873686	3.079484509	-0.140613105
H	2.240578301	2.732313451	0.946453372
H	2.579215127	2.941792921	-0.801688204
H	-4.012500342	-1.943042999	0.435644148
H	-0.583263619	1.435431487	1.211041745
H	-2.845657534	0.518374370	-1.774315562
H	-1.388806396	2.244759975	-1.014076235
H	-2.592799285	2.138866790	0.106952912
O	3.313556782	-1.621483162	-0.780591907
O	2.926493558	-1.125790287	1.539334445
H	2.765563737	-0.759981253	-1.118076171
H	3.183663400	-1.482920387	0.411658442
H	2.538601971	-1.838393831	2.060824640
H	4.231765543	-1.472960734	-1.033527253

x31_32

C	-3.322466343	-0.077216423	0.240433969
C	-2.188913347	0.643393017	-0.494194173
C	-1.284833434	-0.331276973	-1.271790847
S	-0.216347896	-1.379334039	-0.215997048
C	1.411876864	-0.560989798	-0.554090942

C	2.444733210	-0.790525761	0.352223253
C	2.541047613	-1.917739421	1.339079682
O	-3.577114264	0.061783252	1.408551217
O	-4.029248436	-0.874061200	-0.587863874
N	-1.421192018	1.489982421	0.424363209
H	-1.898558420	-0.988877934	-1.883789434
H	-0.599086388	0.216706167	-1.920671125
O	1.515659004	0.231553937	-1.533029212
H	2.951413952	-2.820587833	0.872556805
H	1.566459293	-2.161269475	1.766979430
H	3.207992292	-1.639036615	2.159941378
H	-4.728183210	-1.290685035	-0.061752424
H	-2.670807954	1.287325838	-1.239146606
H	-1.004076281	0.877639657	1.125847220
H	-2.061142055	2.088640961	0.939774500
O	3.722321958	-0.324698692	-0.063657941
H	2.087494898	0.545589379	1.107649556
H	3.598166132	-0.023207278	-0.975626805
O	2.197425214	1.607817916	1.437599946
O	0.959283031	2.552260349	-0.415848509
H	1.637843404	2.156025898	0.596836701
H	1.202546238	1.833965909	-1.059647070
H	3.147688274	1.743782709	1.322522184
H	0.013087383	2.322959170	-0.181641674

x32_33

C	-2.380006854	-0.300120500	-0.272982971
C	-1.159906913	-0.849674249	-1.002263611
C	-0.655437038	-2.131713850	-0.315411137
S	0.220688390	-1.522556617	1.155888901
C	1.009290205	-0.057953879	0.142980344
C	2.355738495	-0.545522361	-0.478694501
C	2.876531134	0.434460514	-1.542605386
O	-2.477779463	0.777898207	0.254066682
O	-3.386147613	-1.194791309	-0.305652629
N	-0.059584968	0.130892627	-1.028954865
H	0.017785253	-2.672685277	-0.986377598
H	-1.477376752	-2.789024375	-0.037760813
O	1.200483358	1.012168582	0.849987242
H	3.907817207	0.171720531	-1.781193041
H	2.861721992	1.446599792	-1.131066129
H	2.306193027	0.419114549	-2.479398513
H	-4.132624928	-0.808596492	0.176167730
H	-1.485516467	-1.063204101	-2.026377100
O	3.294652081	-0.636683921	0.572977769
H	2.250803641	-1.548437854	-0.904931383
H	3.081260781	0.117668346	1.144672564
H	0.089756052	1.812264063	1.491816554

H	0.440508008	-0.003416840	-1.903041772
O	-0.496169628	2.620049292	-0.844307987
O	-0.677066347	2.496100591	1.575618675
H	-0.376731720	1.502741443	-0.970624074
H	-0.685137572	2.676601968	0.255878029
H	-1.470383561	1.970323194	1.735258136
H	0.397352132	2.974943841	-0.941776689

x33_34

C	-2.087069408	0.775075450	0.161745571
C	-1.273571274	0.519970430	-1.112187289
C	-1.272779816	-0.978272057	-1.555181523
S	0.211613797	-1.881006789	-0.963719728
C	0.842932712	0.335775226	-0.010869828
C	2.363430119	0.372538995	-0.174400977
C	2.868850634	1.805500816	0.053222519
O	-2.679573586	-0.066158910	0.803175338
O	-2.149807218	2.079291517	0.446474467
N	0.091060866	1.011111750	-0.957156633
H	-1.280697727	-1.009194579	-2.645734984
H	-2.187260806	-1.453314619	-1.199330383
O	0.401278721	0.288690907	1.202299656
H	3.959005862	1.806021609	0.006383097
H	2.558234813	2.154552126	1.041216485
H	2.475629588	2.498595957	-0.695442399
H	-2.663299260	2.172831788	1.262977400
H	-1.756192643	1.135580548	-1.877040483
O	2.960825476	-0.530217675	0.731502778
H	2.628488046	0.028443885	-1.175936847
H	2.485874439	-0.426228738	1.566605532
H	-0.176844240	-0.752464547	1.619966144
H	0.562659864	1.056382982	-1.852857354
O	-0.721201627	-1.746958679	1.777029308
H	-0.437247816	-2.121126226	0.842223463
H	-1.655528174	-1.494962598	1.654215372

x29+7b_35

C	-0.438015115	0.040230714	1.525157933
C	0.759441968	0.275089325	0.552174537
C	1.215790354	1.735707128	0.582424440
S	2.725108261	2.081603815	-0.418284098
O	-0.347873602	-0.851827436	2.332025507
O	-1.424337452	0.921496129	1.526152697
N	0.533068530	-0.160436034	-0.879735638
H	1.543262327	-0.372809104	0.940868420
H	1.453062351	1.998650631	1.613702885
H	0.423896770	2.398530441	0.239404679
H	2.035854151	2.361233491	-1.546694197

H	-1.607235090	1.315718882	0.617387854
C	-0.437288211	-1.295233700	-1.125880578
C	0.177143016	-2.580697647	-0.515919308
C	-0.733014990	-3.494193689	0.239904017
O	1.364355638	-2.755162333	-0.677963561
O	-1.690518516	-1.047454962	-0.631952777
H	-1.657538833	-3.674760588	-0.312690604
H	-0.213930723	-4.423073270	0.469387238
H	-1.014620876	-2.976293269	1.162983228
H	-0.418929771	-1.419653332	-2.221404684
H	-2.008962335	-0.140541997	-0.893163091
H	1.440466696	-0.498683004	-1.212513526
O	-0.100372191	1.897834577	-2.239160093
O	-2.043328708	1.543032715	-0.911955334
H	0.269738268	0.711546446	-1.549103259
H	-1.163424707	1.857913510	-1.656764941
H	-0.214959535	1.844542944	-3.191818678
H	-2.849419941	2.051069106	-1.031371472

x35_36

C	-2.407919567	1.138190416	-0.170083599
C	-1.690197329	0.088325698	-1.021855541
C	-1.818457049	-1.310662252	-0.385724002
O	-1.879567015	2.082741815	0.371836134
O	-3.730858476	0.916670727	-0.123108032
S	-1.595234139	-1.445873266	1.438383073
N	-0.317913671	0.461066354	-1.372535813
H	-2.255230819	0.047191477	-1.961894059
H	-2.838267094	-1.661526601	-0.541605874
H	-1.142028078	-1.989043097	-0.902641657
H	-4.117353339	1.580774294	0.467606360
H	-0.389723564	-0.842921250	1.529135165
C	0.757503799	0.263802392	-0.465723710
H	-0.311850902	1.381117165	-1.794497561
C	1.479118168	-0.953149179	-0.447807251
C	1.492648763	-1.849642534	-1.665990807
O	2.238244858	-1.278989166	0.515390653
O	0.671398949	0.898688715	0.822034938
H	0.900262269	-1.454737488	-2.488571091
H	1.107966445	-2.830928918	-1.371227396
H	2.528000625	-1.998849179	-1.983027649
H	2.913601466	-0.196264388	1.212585358
H	-0.072338327	1.518374186	0.781292794
O	2.952611092	1.720498128	-0.662645663
O	3.573946119	0.604205909	1.386559110
H	2.053662169	1.056361788	-0.846085568
H	3.331666587	1.253095974	0.337256688
H	2.577703562	2.579133843	-0.431782424

H	4.456989796	0.219405421	1.359912994

x36_34

C	1.412346787	-1.353539705	0.532419759
C	1.539022611	0.166202245	0.810192936
C	2.815309619	0.750709291	0.220133493
O	2.863246840	1.666027141	-0.558552102
O	3.900872868	0.108051098	0.690518399
S	1.196646838	-1.800914989	-1.234518497
N	0.433044944	0.952203222	0.287910675
H	2.320637164	-1.842109737	0.882818091
H	0.572686749	-1.740406922	1.104334078
H	1.599843485	0.292675120	1.896735371
H	4.675975664	0.504154258	0.265537623
H	-0.164623447	-1.646869761	-1.279379820
C	-0.844772856	0.720773491	0.571656560
H	0.607137243	1.479562020	-0.561034870
C	-1.871832760	1.118644701	-0.353222615
C	-3.218407132	1.551886572	0.194170037
O	-1.081573380	-0.018273260	1.630318435
O	-1.377435149	1.947993755	-1.400521756
H	-2.023853615	-0.101557769	-0.885565053
H	-3.598094944	0.846173452	0.933173273
H	-3.938706242	1.593876453	-0.625536359
H	-3.168525205	2.547339035	0.656467583
H	-1.451516555	2.868560657	-1.118439997
O	-2.097770231	-1.367365536	-1.153104167
O	-3.049345747	-1.541680944	1.181308500
H	-2.797194264	-1.641069073	0.192883103
H	-1.947625747	-0.567835196	1.549453328
H	-2.548021989	-1.528549645	-1.987740385
H	-2.970353525	-2.411881592	1.582688720

x29+7b_37

C	1.076757943	1.384988493	-0.263124650
C	0.886914427	0.446520306	0.925499086
C	2.209526281	-0.285785494	1.228509408
S	2.681661968	-1.658908472	0.098822577
O	1.181852014	1.036860711	-1.415609396
O	1.268604747	2.648436513	0.149608922
N	-0.232064075	-0.473856075	0.670683246
H	0.680103910	1.073918600	1.794784664
H	2.134697455	-0.750953870	2.214097030
H	3.011004257	0.453718908	1.279458832
H	2.389423625	-1.015427686	-1.048777518
H	1.421506598	3.188323355	-0.639978731
C	-1.594634897	0.255094316	0.728202706
C	-2.662700907	-0.795743392	0.377362807

H	-3.674071560	-0.345464409	0.310106462
O	-2.466537820	-1.961535008	0.118363753
O	-1.625447302	1.237553912	-0.185655962
C	-1.884161231	0.772476936	2.150214968
H	-1.562490689	0.845288467	-1.563042393
H	-0.206695148	-1.240431480	1.341954538
O	-0.288019180	-1.515595857	-1.628434523
O	-1.389961187	0.458231900	-2.538988868
H	-0.178231673	-1.069787454	-0.566357516
H	-0.778532282	-0.682259031	-2.152666446
H	-0.933878288	-2.226926398	-1.525595332
H	-0.675115109	1.004277682	-2.885728740
H	-2.921338616	1.111561932	2.188582980
H	-1.261017961	1.645517502	2.352182759
H	-1.731362025	0.014072117	2.925816805

x37_38

C	-2.699737449	-0.497447987	0.196462815
C	-1.715462857	-0.314515677	-0.967145448
C	-0.678364622	-1.449745261	-0.922848862
S	0.297206650	-1.373345834	0.628315967
O	-2.796593985	0.190791139	1.181650074
O	-3.487326388	-1.574936593	-0.015183482
N	-1.126458602	1.011662639	-1.113825822
H	-2.312250168	-0.476528931	-1.872850108
H	-0.033015794	-1.338124263	-1.798683059
H	-1.196443679	-2.406614990	-0.991461337
H	-4.045592329	-1.677488510	0.770140683
C	-0.144121776	1.631891849	-0.217641726
C	1.176270150	0.835369588	-0.199140790
C	0.231518517	2.986535254	-0.848041283
H	-1.856407434	1.689233318	-1.295446392
O	-0.588981783	1.943600223	1.079386306
O	2.105871261	1.208150513	0.539863725
H	0.940147609	3.488431212	-0.191028835
H	0.668570401	2.857894075	-1.839881417
H	-0.670270961	3.599078488	-0.925868897
H	-1.123400408	1.206683470	1.406258279
H	1.395412153	0.301438855	-1.138396308
O	4.083558642	-0.239054137	0.196223386
O	2.907358792	-2.312306032	-0.173861179
H	3.519527980	-1.370512666	-0.010298025
H	1.856379646	-2.045114062	0.107894864
H	3.292942562	0.449198376	0.348996362
H	4.621539633	-0.239979383	0.994545450
H	2.929318017	-2.584518236	-1.098375630

x38_30

C	-0.510350134	1.972933561	-0.016029938
C	0.237039385	1.109003094	1.007014168
C	1.751721464	1.237855163	0.852161735
S	2.393367641	0.473404849	-0.683901068
O	0.015277307	2.806022775	-0.699236868
O	-1.847581526	1.808061978	0.068206537
N	-0.212714808	-0.314133897	1.038211809
H	-0.046728024	1.527391823	1.981296919
H	2.246697244	0.788038992	1.714667543
H	2.017385885	2.292286079	0.799067469
H	-2.241720838	2.359922475	-0.622621073
C	0.093580324	-1.129278205	-0.275035564
C	1.635272647	-1.174425661	-0.413434693
C	-0.448733383	-2.547300610	-0.030778257
O	2.138039639	-1.803198778	0.759741278
O	-0.468768634	-0.487132523	-1.313743393
H	1.857429212	-1.743272268	-1.321370774
H	3.099435034	-1.808925256	0.706838930
H	0.004231633	-3.017813997	0.842269354
H	-1.531597415	-2.526837852	0.070636646
H	-0.209236591	-3.143952996	-0.913081163
H	-1.584356944	-0.663644758	-1.299563164
H	0.264660726	-0.782076613	1.809766116
O	-2.731706068	-0.596326091	1.327754933
O	-2.841707476	-0.924116401	-1.073409866
H	-1.344737274	-0.396112050	1.220582803
H	-2.917221856	-0.733904026	0.225477707
H	-3.114989977	0.261583308	1.533611949
H	-3.446760568	-0.395572860	-1.597975057
